# Ammonium tris­(tetra­ethyl­ammonium) hexa­cosa­oxidoocta­molybdate

**DOI:** 10.1107/S1600536808004182

**Published:** 2008-02-15

**Authors:** Ikram Zebiri, Leïla Bencharif, Amani Direm, Mustapha Bencharif, Nourredine Benali-Cherif

**Affiliations:** a Laboratoire de Chimie des Matériaux, Faculté des Sciences, Université Mentouri, 25000, Constantine, Algeria; b Laboratoire des Structures, Propriétés et Interactions Interatomiques, Centre Universitaire de Khenchela, 40000 Khenchela, Algeria

## Abstract

The structure of the title compound, NH_4_(C_8_H_20_N)_3_[Mo_8_O_26_], is built up by discrete cations and anions, with two formula units in the asymmetric unit. The β-octa­molybdate anions are linked to the ammonium cations *via* N—H⋯O hydrogen bonding involving terminal oxide groups and to the tetra­ethyl­ammonium cations *via* weak C—H⋯O inter­actions.

## Related literature

For related literature, see: Böschen *et al.* (1974 ▶); Briceño & Atencio (2004 ▶); Chakrabarti & Natarajan (2002 ▶); Harrison *et al.* (1993 ▶); Hsieh *et al.* (1987 ▶); Lindqvist (1950 ▶); Liu & Guo (2007 ▶); Liu *et al.* (2006 ▶, 2008 ▶); Nelson *et al.* (2006 ▶, 2007 ▶); Niven *et al.* (1991 ▶); Rarig & Zubieta (2001 ▶); Román *et al.* (1994 ▶); Sun *et al.* (2006 ▶); Thorn *et al.* (2005 ▶); Wu *et al.* (2001 ▶); Xiao *et al.* (2005 ▶); Xu *et al.* (2003 ▶); Yan *et al.* (2003 ▶); Zhang *et al.* (2004 ▶).
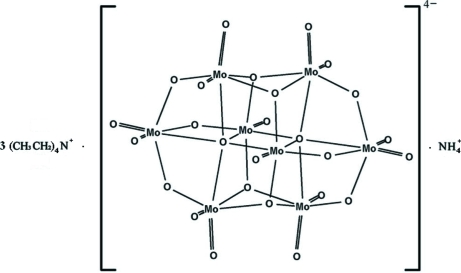


## Experimental

### 

#### Crystal data

NH_4_(C_8_H_20_N)_3_[Mo_8_O_26_]
*M* *_r_* = 1592.31
Monoclinic, 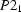

*a* = 10.7154 (2) Å
*b* = 22.8873 (3) Å
*c* = 19.9338 (2) Å
β = 96.906 (8)°
*V* = 4853.23 (12) Å^3^
*Z* = 4
Mo *K*α radiation
μ = 2.08 mm^−1^
*T* = 293 (2) K
0.25 × 0.15 × 0.05 mm

#### Data collection

Nonius KappaCCD diffractometer
Absorption correction: none
57681 measured reflections
29087 independent reflections
21795 reflections with *I* > 2σ(*I*)
*R*_int_ = 0.074

#### Refinement

*R*[*F* ^2^ > 2σ(*F* ^2^)] = 0.048
*wR*(*F* ^2^) = 0.124
*S* = 1.01
29087 reflections
1117 parameters
1 restraint
H-atom parameters constrained
Δρ_max_ = 0.86 e Å^−3^
Δρ_min_ = −1.29 e Å^−3^
Absolute structure: Flack (1983 ▶), with 13317 Friedel pairs
Flack parameter: 0.17 (4)

### 

Data collection: *KappaCCD Server Software* (Nonius, 1998 ▶); cell refinement: *DENZO* and *SCALEPACK* (Otwinowski & Minor, 1997 ▶); data reduction: *DENZO* and *SCALEPACK*; program(s) used to solve structure: *SIR2004* (Burla *et al.*, 2005 ▶); program(s) used to refine structure: *SHELXL97* (Sheldrick, 2008 ▶); molecular graphics: *ORTEP-3* (Farrugia, 1997 ▶) and *PLATON* (Spek, 2003 ▶); software used to prepare material for publication: *WinGX* (Farrugia, 1999 ▶).

## Supplementary Material

Crystal structure: contains datablocks global, I. DOI: 10.1107/S1600536808004182/bt2676sup1.cif

Structure factors: contains datablocks I. DOI: 10.1107/S1600536808004182/bt2676Isup2.hkl

Additional supplementary materials:  crystallographic information; 3D view; checkCIF report

## Figures and Tables

**Table 1:** Hydrogen-bond geometry (Å, °)

*D*—H⋯*A*	*D*—H	H⋯*A*	*D*⋯*A*	*D*—H⋯*A*
N4*A*—H1N⋯O12*B*	0.82	2.399 (6)	2.908 (10)	121.32 (58)
N4*A*—H1N⋯O21*B*	0.82	2.388 (5)	2.851 (10)	116.76 (59)
N4*A*—H1N⋯O32*B*	0.82	2.571 (5)	2.936 (9)	108.68 (57)
N4*A*—H1N⋯O71*B*	0.82	2.482 (5)	2.904 (10)	113.29 (55)
N4*A*—H2N⋯O42*A*	0.96	2.457 (5)	2.856 (10)	104.87 (51)
N4*A*—H2N⋯O51*A*	0.96	2.216 (5)	2.915 (10)	129.21 (53)
N4*A*—H3N⋯O12*B*	0.73	2.566 (6)	2.908 (10)	110.92 (67)
N4*A*—H3N⋯O62*A*	0.73	2.261 (5)	2.909 (9)	148.30 (5)
N4*A*—H4N⋯O42*A*	0.96	2.511 (6)	2.856 (10)	101.14 (51)
N4*A*—H4N⋯O81*A*	0.96	1.999 (5)	2.865 (10)	149.24 (50)
N4*B*—H5N⋯O12*A*^i^	0.84	2.441 (6)	2.854 (10)	110.89 (56)
N4*B*—H5N⋯O32*A*^i^	0.84	2.445 (5)	2.950 (9)	119.12 (54)
N4*B*—H5N⋯O71*A*^i^	0.84	2.458 (6)	2.943 (10)	117.38 (53)
N4*B*—H6N⋯O12*A*^i^	0.95	2.387 (5)	2.854 (10)	109.94 (50)
N4*B*—H6N⋯O62*B*	0.95	2.214 (5)	2.992 (9)	138.49 (51)
N4*B*—H7N⋯O42*B*	0.97	2.385 (6)	2.820 (10)	106.78 (50)
N4*B*—H7N⋯O81*B*	0.97	2.089 (5)	2.895 (10)	139.63 (48)
N4*B*—H8N⋯O51*B*	0.96	2.122 (5)	2.855 (10)	132.53 (52)

Symmetry code: (i) 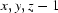.

**Fig. 1.**
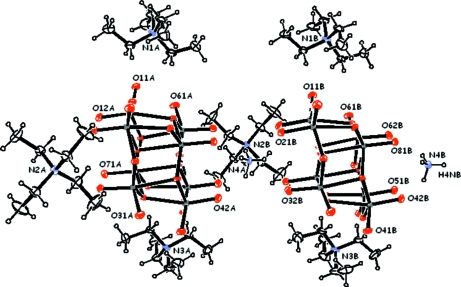
Asymmetric unit of the title compound, showing 30% probability displacement ellipsoids and arbitrary spheres for H atoms.

**Fig. 2.**
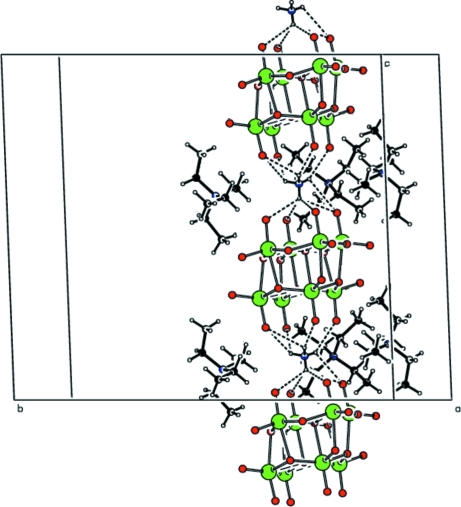
A partial packing diagram of the title compound with N—H···O hydrogen bonds shown as dashed lines.

## supplementary crystallographic information

### Comment

The physical properties of polyoxomolybdates, containing Mo(VI) have made them the subject of intense research works for many years (Lindqvist, 1950; Román *et al.*, 1994; Yan *et al.*, 2003; Zhang *et al.*, 2004; Liu *et al.*, 2006; Nelson *et al.*, 2007). These materials are generally constructed from [MoO*_x_*] polyhedra sharing common vertices and edges with one another and forming large anionic architectures (Briceño & Atencio, 2004; Thorn *et al.*, 2005).

The octamolybdates have been the focus of special attention owing to the presence of several structure types (Hsieh *et al.*, 1987; Harrison *et al.*, 1993; Rarig & Zubieta, 2001; Niven *et al.*, 1991; Nelson *et al.*, 2006). Several compounds consisting of octamolybdate anions have been reported (Böschen *et al.*, 1974; Chakrabarti & Natarajan, 2002; Xu *et al.*, 2003; Xiao *et al.*, 2005).

The asymmetric unit of the title compound consists of two ammonium, six tetraethylammonium cations and two hexacosaoxaoctamolybdate anions (Fig. 1).

The structure of the anions, as described recently (Liu & Guo, 2007), is constructed from an array of eight edge-shared MoO_6_ octahedra with 14 O(*t*), six O(µ2), four O(µ3) and two O(µ5) atoms.

The Mo—O distances, ranging from 1.684 (1) to 2.478 (1) Å, agree with those reported for other [Mo_8_O_26_]^4-^ anions in the literature (Liu *et al.*, 2008; Sun *et al.*, 2006; Wu *et al.*, 2001) and can be grouped into four sets bridging groups [Mo—O(terminal) 1.684 (1)–1.720 (1) Å, Mo—O(µ2): 1.742 (1)–2.271 (1) Å, Mo—O(µ3): 1.938 (1)–2.336 (1) Å and Mo—O(µ5): 2.108 (1)–2.477 (1) Å.

Polyhedral octamolybdates are separated by ammonium cations (Fig. 2) creating chains running along the *c* axis. In the crystal structure, an extended array of weak intermolecular C—H···O hydrogen bonds stabilizes the crystal packing.

### Experimental

Single crystals of the title compound are prepared from a mixture of (NH_4_)_6_Mo_7_O_24_.1.5H_2_O (137 mg, 1 mmol), (C_2_H_5_)_4_NCl.H_2_O (386 mg, 3 mmol) and 3 ml H_2_O, heated in a Teflon-lined steel autoclave inside a programmable electric furnace at 160°C for 2 days. After cooling the autoclave to room temperature for 48 h, colorless crystals were obtained, filtered, washed with H_2_O, EtOH, Et_2_OH and dried in air.

### Refinement

The title compound crystallizes in the non centrosymmetric space group *P*2_1_ but the structure shows pseudo-symmetry m, which is fulfilled for approximately 91% of the atoms. Refinement in a higher symmetric space group is not possible. H atoms bonded to C were refined as riding with C—H distances of 0.97 Å or 0.96 Å and *U*_iso_(H) = 1.2*U*_eq_(C) or *U*_iso_(H) = 1.5*U*_eq_(C), for CH_2_ and CH_3_ groups respectively. Hydrogen atoms of the ammonium cations were located in difference Fourier syntheses and refined as riding with *U*_iso_(H) = 0.036 Å^2^.

### Crystal data

**Table:**

NH_4_(C_8_H_20_N)_3_[Mo_8_O_26_]	*F*_000_ = 3120
*M**_r_* = 1592.31	*D*_x_ = 2.179 Mg m^−^^3^
Monoclinic, *P*2_1_	Mo *K*α radiation λ = 0.71073 Å
Hall symbol: P 2yb	Cell parameters from 29087 reflections
*a* = 10.7154 (2) Å	θ = 1.0–31.0º
*b* = 22.8873 (3) Å	µ = 2.08 mm^−^^1^
*c* = 19.9338 (2) Å	*T* = 293 (2) K
β = 96.906 (8)º	Prism, colourless
*V* = 4853.23 (12) Å^3^	0.25 × 0.15 × 0.05 mm
*Z* = 4	

### Data collection

**Table:**

Nonius KappaCCD diffractometer	21795 reflections with *I* > 2σ(*I*)
Radiation source: fine-focus sealed tube	*R*_int_ = 0.074
Monochromator: graphite	θ_max_ = 31.0º
*T* = 293(2) K	θ_min_ = 1.0º
ω/θ scans	*h* = −15→12
Absorption correction: none	*k* = −31→33
57681 measured reflections	*l* = −28→25
29087 independent reflections	

### Refinement

**Table:**

Refinement on *F*^2^	Hydrogen site location: difference Fourier map
Least-squares matrix: full	H-atom parameters constrained
*R*[*F*^2^ > 2σ(*F*^2^)] = 0.048	*w* = 1/[σ^2^(*F*_o_^2^) + (0.0616*P*)^2^] where *P* = (*F*_o_^2^ + 2*F*_c_^2^)/3
*wR*(*F*^2^) = 0.124	(Δ/σ)_max_ = 0.003
*S* = 1.01	Δρ_max_ = 0.86 e Å^−^^3^
29087 reflections	Δρ_min_ = −1.29 e Å^−^^3^
1117 parameters	Extinction correction: none
1 restraint	Absolute structure: Flack (1983), with 13317 Friedel pairs
Primary atom site location: structure-invariant direct methods	Flack parameter: 0.17 (4)
Secondary atom site location: difference Fourier map	

### Special details

**Table:**

Geometry. All e.s.d.'s (except the e.s.d. in the dihedral angle between two l.s. planes) are estimated using the full covariance matrix. The cell e.s.d.'s are taken into account individually in the estimation of e.s.d.'s in distances, angles and torsion angles; correlations between e.s.d.'s in cell parameters are only used when they are defined by crystal symmetry. An approximate (isotropic) treatment of cell e.s.d.'s is used for estimating e.s.d.'s involving l.s. planes.
Refinement. Refinement of *F*^2^ against ALL reflections. The weighted *R*-factor *wR* and goodness of fit *S* are based on *F*^2^, conventional *R*-factors *R* are based on *F*, with *F* set to zero for negative *F*^2^. The threshold expression of *F*^2^ > σ(*F*^2^) is used only for calculating *R*-factors(gt) *etc*. and is not relevant to the choice of reflections for refinement. *R*-factors based on *F*^2^ are statistically about twice as large as those based on *F*, and *R*- factors based on ALL data will be even larger.

### Fractional atomic coordinates and isotropic or equivalent isotropic displacement parameters (Å^2^)

**Table:**

	*x*	*y*	*z*	*U*_iso_*/*U*_eq_	
C11B	0.5900 (10)	−0.0413 (4)	−1.1150 (6)	0.066 (3)	
H11F	0.6002	−0.0562	−1.1596	0.080*	
H11G	0.5827	−0.0747	−1.0858	0.080*	
C12B	0.4705 (11)	−0.0079 (6)	−1.1198 (6)	0.090 (4)	
H12F	0.4019	−0.0329	−1.1367	0.135*	
H12H	0.4746	0.0245	−1.1500	0.135*	
H12G	0.4576	0.0063	−1.0759	0.135*	
C17B	0.8094 (10)	−0.0531 (4)	−1.0826 (6)	0.062 (3)	
H17G	0.8087	−0.0722	−1.1261	0.074*	
H17F	0.7886	−0.0824	−1.0507	0.074*	
C18B	0.9424 (11)	−0.0322 (5)	−1.0604 (7)	0.089 (4)	
H18H	0.9992	−0.0647	−1.0593	0.134*	
H18F	0.9466	−0.0152	−1.0161	0.134*	
H18G	0.9655	−0.0034	−1.0917	0.134*	
C13B	0.6950 (9)	0.0206 (4)	−1.0217 (5)	0.051 (2)	
H13G	0.7733	0.0405	−1.0069	0.061*	
H13F	0.6298	0.0501	−1.0289	0.061*	
C14B	0.6647 (11)	−0.0188 (5)	−0.9654 (6)	0.072 (3)	
H14H	0.6587	0.0040	−0.9255	0.108*	
H14F	0.7300	−0.0474	−0.9562	0.108*	
H14G	0.5860	−0.0382	−0.9786	0.108*	
C15B	0.7342 (10)	0.0402 (4)	−1.1364 (4)	0.056 (3)	
H15F	0.6614	0.0657	−1.1421	0.067*	
H15G	0.8039	0.0629	−1.1145	0.067*	
C16B	0.7648 (12)	0.0237 (6)	−1.2051 (5)	0.081 (3)	
H16F	0.7765	0.0585	−1.2306	0.121*	
H16H	0.6971	0.0012	−1.2279	0.121*	
H16G	0.8407	0.0010	−1.2008	0.121*	
C37A	0.8065 (8)	0.5109 (4)	−0.7215 (4)	0.0412 (19)	
H37C	0.8783	0.4851	−0.7117	0.049*	
H37D	0.7340	0.4867	−0.7359	0.049*	
C38A	0.8300 (11)	0.5503 (5)	−0.7793 (5)	0.069 (3)	
H38C	0.8431	0.5269	−0.8179	0.103*	
H38D	0.9032	0.5737	−0.7663	0.103*	
H38E	0.7586	0.5752	−0.7906	0.103*	
C33A	0.6717 (9)	0.5826 (4)	−0.6681 (5)	0.063 (3)	
H33C	0.6629	0.6025	−0.6259	0.076*	
H33D	0.6894	0.6122	−0.7006	0.076*	
C34A	0.5496 (10)	0.5544 (5)	−0.6928 (6)	0.079 (3)	
H34E	0.4855	0.5838	−0.7007	0.119*	
H34D	0.5274	0.5273	−0.6595	0.119*	
H34C	0.5572	0.5340	−0.7342	0.119*	
C31A	0.7639 (10)	0.4937 (4)	−0.6057 (4)	0.055 (2)	
H31C	0.6935	0.4697	−0.6242	0.066*	
H31D	0.8379	0.4690	−0.5998	0.066*	
C32A	0.7386 (13)	0.5163 (6)	−0.5368 (5)	0.095 (4)	
H32C	0.7253	0.4839	−0.5080	0.143*	
H32D	0.6652	0.5407	−0.5420	0.143*	
H32E	0.8095	0.5387	−0.5171	0.143*	
C35A	0.8932 (10)	0.5803 (4)	−0.6292 (5)	0.068 (3)	
H35C	0.8783	0.5944	−0.5850	0.081*	
H35D	0.8951	0.6140	−0.6586	0.081*	
C36A	1.0221 (10)	0.5509 (5)	−0.6228 (6)	0.093 (4)	
H36D	1.0853	0.5783	−0.6049	0.139*	
H36E	1.0396	0.5380	−0.6665	0.139*	
H36C	1.0224	0.5180	−0.5930	0.139*	
C31B	0.7787 (12)	0.4874 (5)	−1.1157 (5)	0.073 (3)	
H31F	0.7046	0.4664	−1.1355	0.088*	
H31G	0.8496	0.4609	−1.1146	0.088*	
C32B	0.7615 (14)	0.5032 (7)	−1.0450 (7)	0.117 (5)	
H32F	0.7463	0.4685	−1.0202	0.176*	
H32G	0.6912	0.5292	−1.0452	0.176*	
H32H	0.8360	0.5222	−1.0239	0.176*	
C36B	1.0417 (10)	0.5365 (6)	−1.1401 (6)	0.090 (4)	
H36G	1.1121	0.5606	−1.1236	0.135*	
H36H	1.0488	0.5250	−1.1858	0.135*	
H36F	1.0408	0.5023	−1.1123	0.135*	
C35B	0.9245 (10)	0.5695 (6)	−1.1379 (7)	0.099 (4)	
H35F	0.9298	0.6047	−1.1645	0.119*	
H35G	0.9212	0.5816	−1.0916	0.119*	
C33B	0.7009 (11)	0.5859 (5)	−1.1646 (7)	0.082 (4)	
H33F	0.7056	0.6041	−1.1204	0.099*	
H33G	0.7190	0.6157	−1.1965	0.099*	
C34B	0.5712 (10)	0.5651 (6)	−1.1835 (8)	0.101 (5)	
H34H	0.5144	0.5977	−1.1852	0.152*	
H34G	0.5497	0.5375	−1.1505	0.152*	
H34F	0.5649	0.5467	−1.2270	0.152*	
C38B	0.8294 (12)	0.5536 (6)	−1.2869 (6)	0.099 (5)	
H38F	0.8290	0.5321	−1.3283	0.149*	
H38G	0.9102	0.5715	−1.2755	0.149*	
H38H	0.7657	0.5832	−1.2924	0.149*	
C37B	0.8032 (9)	0.5122 (4)	−1.2306 (5)	0.057 (3)	
H37G	0.7225	0.4936	−1.2436	0.069*	
H37F	0.8666	0.4817	−1.2268	0.069*	
C26B	0.6949 (9)	0.3370 (6)	−0.9758 (6)	0.098 (5)	
H26H	0.6245	0.3564	−1.0007	0.148*	
H26F	0.7330	0.3112	−1.0055	0.148*	
H26G	0.7554	0.3655	−0.9576	0.148*	
C25B	0.6511 (6)	0.3026 (3)	−0.9195 (3)	0.0489 (17)	
H25G	0.5889	0.2746	−0.9389	0.059*	
H25F	0.6091	0.3291	−0.8916	0.059*	
C27B	0.6876 (7)	0.2404 (4)	−0.8198 (4)	0.061 (2)	
H27F	0.6205	0.2161	−0.8415	0.073*	
H27G	0.6492	0.2706	−0.7949	0.073*	
C28B	0.7712 (11)	0.2031 (7)	−0.7696 (6)	0.109 (5)	
H28G	0.7218	0.1865	−0.7373	0.163*	
H28F	0.8367	0.2269	−0.7466	0.163*	
H28H	0.8080	0.1722	−0.7934	0.163*	
C21B	0.8170 (7)	0.2264 (4)	−0.9148 (4)	0.056 (2)	
H21F	0.8832	0.2068	−0.8858	0.067*	
H21G	0.8561	0.2476	−0.9489	0.067*	
C22B	0.7296 (10)	0.1811 (7)	−0.9492 (7)	0.118 (5)	
H22G	0.7759	0.1555	−0.9754	0.177*	
H22H	0.6642	0.2001	−0.9784	0.177*	
H22F	0.6931	0.1587	−0.9157	0.177*	
C23B	0.8546 (6)	0.3111 (4)	−0.8435 (4)	0.0550 (19)	
H23F	0.9185	0.2889	−0.8159	0.066*	
H23G	0.8939	0.3282	−0.8803	0.066*	
C25A	−0.1879 (7)	0.2949 (4)	−0.4261 (4)	0.065 (2)	
H25D	−0.1448	0.2662	−0.4508	0.078*	
H25C	−0.1250	0.3213	−0.4039	0.078*	
C26A	−0.2785 (12)	0.3297 (7)	−0.4761 (7)	0.124 (6)	
H26E	−0.2325	0.3484	−0.5086	0.186*	
H26D	−0.3201	0.3588	−0.4521	0.186*	
H26C	−0.3399	0.3038	−0.4990	0.186*	
C27A	−0.1540 (6)	0.2306 (4)	−0.3304 (3)	0.0507 (18)	
H27D	−0.0903	0.2578	−0.3109	0.061*	
H27C	−0.1143	0.2040	−0.3593	0.061*	
C28A	−0.2019 (11)	0.1955 (6)	−0.2736 (6)	0.092 (4)	
H28D	−0.1328	0.1756	−0.2482	0.139*	
H28E	−0.2627	0.1674	−0.2925	0.139*	
H28C	−0.2404	0.2215	−0.2443	0.139*	
C21A	−0.3582 (7)	0.2250 (4)	−0.4049 (4)	0.059 (2)	
H21C	−0.4010	0.2079	−0.3694	0.070*	
H21D	−0.4185	0.2492	−0.4324	0.070*	
C22A	−0.3181 (9)	0.1771 (6)	−0.4477 (7)	0.114 (5)	
H22D	−0.3903	0.1548	−0.4659	0.171*	
H22C	−0.2600	0.1521	−0.4209	0.171*	
H22E	−0.2782	0.1933	−0.4840	0.171*	
C23A	−0.3159 (7)	0.3067 (4)	−0.3295 (4)	0.0575 (19)	
H23C	−0.3591	0.2848	−0.2976	0.069*	
H23D	−0.3791	0.3281	−0.3586	0.069*	
C15A	0.7166 (12)	0.0417 (4)	−0.6414 (5)	0.070 (3)	
H15C	0.6406	0.0651	−0.6455	0.084*	
H15D	0.7853	0.0669	−0.6232	0.084*	
C16A	0.7398 (15)	0.0239 (6)	−0.7106 (6)	0.111 (5)	
H16C	0.7429	0.0580	−0.7384	0.167*	
H16E	0.6732	−0.0012	−0.7298	0.167*	
H16D	0.8184	0.0034	−0.7083	0.167*	
C11A	0.5875 (13)	−0.0434 (4)	−0.6125 (7)	0.089 (4)	
H11C	0.5995	−0.0625	−0.6546	0.106*	
H11D	0.5840	−0.0738	−0.5788	0.106*	
C12A	0.4644 (12)	−0.0134 (7)	−0.6217 (6)	0.101 (4)	
H12C	0.3991	−0.0414	−0.6343	0.152*	
H12E	0.4643	0.0156	−0.6566	0.152*	
H12D	0.4498	0.0052	−0.5802	0.152*	
C13A	0.6887 (11)	0.0267 (5)	−0.5263 (5)	0.064 (3)	
H13D	0.6175	0.0530	−0.5348	0.077*	
H13C	0.7630	0.0506	−0.5147	0.077*	
C14A	0.6698 (12)	−0.0111 (5)	−0.4652 (6)	0.087 (4)	
H14E	0.6620	0.0136	−0.4270	0.131*	
H14C	0.7407	−0.0366	−0.4552	0.131*	
H14D	0.5948	−0.0340	−0.4751	0.131*	
C17A	0.8105 (12)	−0.0467 (6)	−0.5868 (8)	0.095 (4)	
H17D	0.8079	−0.0670	−0.6296	0.114*	
H17C	0.7995	−0.0756	−0.5525	0.114*	
C18A	0.9391 (14)	−0.0192 (7)	−0.5705 (8)	0.128 (6)	
H18E	1.0026	−0.0488	−0.5711	0.192*	
H18C	0.9455	−0.0017	−0.5265	0.192*	
H18D	0.9507	0.0101	−0.6035	0.192*	
C24B	0.8107 (10)	0.3601 (6)	−0.8011 (7)	0.120 (6)	
H24F	0.8811	0.3842	−0.7846	0.180*	
H24H	0.7741	0.3439	−0.7635	0.180*	
H24G	0.7492	0.3832	−0.8281	0.180*	
C24A	−0.2284 (10)	0.3512 (6)	−0.2896 (7)	0.106 (4)	
H24C	−0.2771	0.3764	−0.2642	0.159*	
H24D	−0.1867	0.3742	−0.3204	0.159*	
H24E	−0.1670	0.3309	−0.2592	0.159*	
O63A	0.4350 (4)	0.2364 (2)	−0.5727 (2)	0.0240 (10)	
O63B	0.4454 (4)	0.2441 (2)	−1.0729 (2)	0.0251 (10)	
O33A	0.0596 (4)	0.3021 (2)	−0.6769 (2)	0.0293 (11)	
O84B	0.0628 (5)	0.1691 (2)	−1.1462 (2)	0.0319 (11)	
O33B	0.0529 (4)	0.2868 (2)	−1.1749 (2)	0.0277 (11)	
O82A	0.2989 (5)	0.2895 (2)	−0.6797 (2)	0.0260 (11)	
O83A	0.2917 (4)	0.1778 (2)	−0.6589 (2)	0.0273 (10)	
O83B	0.3178 (5)	0.1763 (2)	−1.1557 (2)	0.0275 (10)	
O72A	0.1962 (4)	0.2486 (2)	−0.5683 (2)	0.0236 (11)	
O82B	0.2924 (5)	0.2873 (2)	−1.1802 (2)	0.0244 (10)	
O21A	0.0558 (5)	0.1961 (2)	−0.4558 (2)	0.0399 (13)	
O74B	0.4374 (4)	0.3622 (2)	−1.1003 (2)	0.0291 (10)	
O22A	−0.0928 (5)	0.1334 (2)	−0.5480 (3)	0.0475 (13)	
O42B	0.1132 (5)	0.3393 (3)	−1.2918 (2)	0.0411 (13)	
O73A	0.2035 (5)	0.3600 (2)	−0.5897 (2)	0.0263 (10)	
O72B	0.2058 (4)	0.2430 (2)	−1.0658 (2)	0.0225 (10)	
O51A	0.4373 (5)	0.3422 (3)	−0.7930 (3)	0.0415 (13)	
O73B	0.1823 (4)	0.3543 (2)	−1.0915 (2)	0.0246 (9)	
O32B	0.0710 (5)	0.3227 (3)	−0.9782 (2)	0.0373 (13)	
O41A	0.0695 (5)	0.4257 (2)	−0.6961 (3)	0.0434 (13)	
O31B	−0.0832 (5)	0.3561 (2)	−1.0875 (2)	0.0443 (13)	
O52A	0.5870 (5)	0.4049 (2)	−0.7032 (3)	0.0484 (14)	
O84A	0.0366 (4)	0.1842 (2)	−0.6525 (2)	0.0319 (11)	
O13A	0.1705 (4)	0.1343 (2)	−0.5490 (2)	0.0324 (11)	
O22B	−0.0431 (5)	0.1136 (2)	−1.0348 (3)	0.0478 (14)	
O43A	0.3238 (5)	0.4046 (2)	−0.6998 (2)	0.0328 (11)	
O23A	−0.0547 (5)	0.2549 (2)	−0.5672 (2)	0.0322 (11)	
O62B	0.4271 (5)	0.2088 (3)	−1.2696 (3)	0.0417 (14)	
O42A	0.1404 (5)	0.3521 (3)	−0.7901 (2)	0.0393 (13)	
O81B	0.1379 (5)	0.2055 (2)	−1.2653 (2)	0.0328 (12)	
O11A	0.4260 (5)	0.1128 (2)	−0.5524 (3)	0.0423 (13)	
O61A	0.5559 (5)	0.1622 (2)	−0.6610 (3)	0.0455 (13)	
O62A	0.4122 (5)	0.2059 (2)	−0.7701 (3)	0.0391 (13)	
O12B	0.3875 (5)	0.1922 (3)	−0.9539 (2)	0.0391 (12)	
O52B	0.5442 (5)	0.4169 (2)	−1.2100 (3)	0.0456 (13)	
O41B	0.0283 (5)	0.4094 (2)	−1.1990 (3)	0.0432 (13)	
O71A	0.3722 (5)	0.3209 (3)	−0.4789 (2)	0.0362 (12)	
O71B	0.3627 (5)	0.3265 (2)	−0.9816 (2)	0.0328 (12)	
O32A	0.0814 (5)	0.3313 (3)	−0.4784 (3)	0.0393 (13)	
O51B	0.4086 (5)	0.3439 (3)	−1.2959 (3)	0.0433 (13)	
O53A	0.5489 (4)	0.2832 (2)	−0.6818 (3)	0.0306 (11)	
O74A	0.4590 (5)	0.3546 (2)	−0.5951 (2)	0.0301 (10)	
O23B	−0.0423 (5)	0.2361 (2)	−1.0614 (2)	0.0318 (11)	
O12A	0.3559 (5)	0.1859 (3)	−0.4581 (2)	0.0406 (13)	
O53B	0.5426 (4)	0.2943 (2)	−1.1851 (3)	0.0317 (11)	
O61B	0.5830 (5)	0.1748 (3)	−1.1601 (3)	0.0466 (13)	
O81A	0.1240 (5)	0.2178 (2)	−0.7693 (2)	0.0363 (12)	
O31A	−0.0594 (5)	0.3763 (2)	−0.5860 (3)	0.0469 (14)	
O11B	0.4731 (5)	0.1207 (2)	−1.0460 (3)	0.0448 (14)	
O21B	0.0913 (5)	0.1883 (2)	−0.9499 (2)	0.0374 (12)	
O13B	0.2153 (5)	0.1291 (2)	−1.0418 (2)	0.0343 (11)	
O43B	0.2849 (5)	0.4023 (2)	−1.2039 (2)	0.0342 (11)	
Mo8B	0.16071 (6)	0.21879 (2)	−1.18123 (3)	0.02338 (12)	
Mo6B	0.46244 (6)	0.22070 (3)	−1.18465 (3)	0.02825 (13)	
Mo8A	0.14772 (6)	0.22861 (2)	−0.68488 (3)	0.02326 (13)	
Mo7A	0.34782 (6)	0.30996 (3)	−0.56384 (3)	0.02454 (12)	
Mo7B	0.33886 (6)	0.31190 (2)	−1.06563 (3)	0.02297 (12)	
Mo5B	0.44034 (6)	0.36111 (3)	−1.21243 (3)	0.03065 (14)	
Mo3A	0.04691 (6)	0.32317 (3)	−0.56325 (3)	0.02823 (14)	
Mo3B	0.03669 (6)	0.31022 (3)	−1.06231 (3)	0.02674 (13)	
Mo2A	0.02633 (6)	0.18241 (3)	−0.54103 (3)	0.02875 (13)	
Mo2B	0.05973 (6)	0.17012 (3)	−1.03372 (3)	0.02931 (13)	
Mo6A	0.44880 (6)	0.21524 (2)	−0.68482 (3)	0.02695 (13)	
Mo4A	0.16441 (6)	0.36812 (2)	−0.70606 (3)	0.02810 (13)	
Mo5A	0.46756 (6)	0.35566 (3)	−0.70857 (3)	0.02927 (13)	
Mo4B	0.13666 (6)	0.35758 (2)	−1.20848 (3)	0.02786 (13)	
Mo1A	0.33042 (6)	0.17053 (2)	−0.54277 (3)	0.02758 (13)	
Mo1B	0.36327 (6)	0.17345 (2)	−1.03793 (3)	0.02745 (13)	
N1B	0.7074 (7)	−0.0082 (3)	−1.0889 (4)	0.0454 (17)	
N3A	0.7839 (7)	0.5418 (3)	−0.6566 (4)	0.0444 (17)	
N3B	0.8013 (8)	0.5396 (3)	−1.1623 (4)	0.059 (2)	
N2B	0.7529 (7)	0.2690 (4)	−0.8734 (4)	0.0378 (10)	
N2A	−0.2534 (7)	0.2637 (4)	−0.3725 (4)	0.0391 (12)	
N1A	0.7031 (8)	−0.0053 (3)	−0.5909 (4)	0.060 (2)	
N4A	0.2511 (8)	0.2697 (4)	−0.8729 (4)	0.0364 (10)	
N4B	0.2417 (8)	0.2655 (4)	−1.3738 (4)	0.0364 (11)	
H1N	0.2450	0.2606	−0.9128	0.036*	
H2N	0.2805	0.3089	−0.8656	0.036*	
H3N	0.3051	0.2519	−0.8596	0.036*	
H4N	0.1832	0.2583	−0.8486	0.036*	
H5N	0.2301	0.2638	−1.4164	0.036*	
H6N	0.3010	0.2356	−1.3606	0.036*	
H7N	0.1723	0.2543	−1.3497	0.036*	
H8N	0.2696	0.3050	−1.3668	0.036*	

### Atomic displacement parameters (Å^2^)

**Table:**

	*U*^11^	*U*^22^	*U*^33^	*U*^12^	*U*^13^	*U*^23^
C11B	0.075 (6)	0.034 (4)	0.085 (6)	−0.012 (4)	−0.015 (5)	−0.015 (4)
C12B	0.076 (8)	0.092 (9)	0.096 (8)	−0.023 (7)	−0.014 (6)	−0.024 (7)
C17B	0.069 (6)	0.033 (4)	0.085 (7)	0.016 (4)	0.018 (5)	−0.010 (4)
C18B	0.063 (6)	0.053 (6)	0.153 (11)	0.026 (5)	0.015 (7)	−0.013 (7)
C13B	0.054 (6)	0.039 (5)	0.056 (5)	0.005 (4)	−0.002 (4)	0.001 (4)
C14B	0.077 (8)	0.052 (6)	0.091 (8)	−0.009 (6)	0.028 (6)	0.007 (5)
C15B	0.083 (7)	0.031 (4)	0.052 (5)	−0.012 (4)	0.000 (4)	−0.011 (4)
C16B	0.124 (9)	0.057 (6)	0.061 (6)	−0.003 (6)	0.010 (6)	−0.005 (5)
C37A	0.047 (5)	0.022 (3)	0.054 (4)	0.000 (3)	0.001 (4)	−0.012 (3)
C38A	0.075 (7)	0.063 (6)	0.065 (6)	−0.012 (6)	−0.003 (5)	0.013 (5)
C33A	0.069 (6)	0.037 (5)	0.078 (6)	0.024 (4)	−0.011 (5)	−0.012 (4)
C34A	0.055 (6)	0.073 (7)	0.103 (8)	0.025 (5)	−0.017 (5)	−0.012 (6)
C31A	0.072 (6)	0.039 (5)	0.052 (5)	0.008 (4)	0.002 (4)	−0.001 (4)
C32A	0.144 (11)	0.087 (9)	0.056 (6)	0.000 (9)	0.018 (7)	−0.012 (6)
C35A	0.080 (7)	0.040 (5)	0.076 (6)	−0.014 (5)	−0.019 (5)	−0.008 (4)
C36A	0.070 (7)	0.065 (7)	0.131 (10)	−0.012 (6)	−0.042 (7)	0.011 (7)
C31B	0.097 (8)	0.038 (5)	0.080 (7)	0.023 (5)	−0.012 (6)	−0.013 (5)
C32B	0.125 (12)	0.122 (12)	0.103 (10)	0.034 (10)	0.006 (8)	−0.008 (9)
C36B	0.060 (6)	0.081 (8)	0.121 (9)	0.010 (6)	−0.019 (6)	−0.040 (7)
C35B	0.068 (7)	0.076 (8)	0.143 (11)	0.004 (6)	−0.030 (7)	−0.041 (7)
C33B	0.086 (8)	0.034 (5)	0.127 (10)	0.013 (5)	0.011 (7)	0.000 (6)
C34B	0.054 (6)	0.064 (7)	0.184 (14)	0.010 (5)	0.004 (7)	0.027 (8)
C38B	0.100 (10)	0.081 (9)	0.107 (9)	0.009 (8)	−0.025 (7)	0.048 (7)
C37B	0.058 (6)	0.035 (5)	0.072 (6)	0.011 (4)	−0.023 (5)	−0.013 (4)
C26B	0.046 (5)	0.133 (12)	0.114 (9)	−0.022 (6)	−0.003 (5)	0.059 (8)
C25B	0.031 (3)	0.065 (5)	0.048 (4)	−0.001 (3)	−0.004 (3)	0.010 (4)
C27B	0.043 (4)	0.084 (7)	0.061 (5)	0.013 (4)	0.024 (4)	0.016 (4)
C28B	0.081 (8)	0.162 (13)	0.091 (8)	0.057 (8)	0.040 (6)	0.079 (8)
C21B	0.040 (4)	0.060 (5)	0.071 (5)	−0.003 (4)	0.022 (3)	−0.023 (4)
C22B	0.072 (8)	0.110 (10)	0.176 (12)	−0.011 (8)	0.027 (8)	−0.087 (10)
C23B	0.032 (4)	0.065 (5)	0.064 (5)	0.009 (4)	−0.008 (3)	−0.009 (4)
C25A	0.043 (4)	0.091 (7)	0.067 (5)	0.015 (4)	0.028 (4)	0.025 (5)
C26A	0.084 (8)	0.170 (15)	0.127 (11)	0.045 (9)	0.051 (8)	0.094 (10)
C27A	0.032 (4)	0.066 (5)	0.053 (4)	0.006 (3)	0.005 (3)	−0.007 (4)
C28A	0.087 (8)	0.108 (10)	0.079 (7)	0.007 (7)	0.001 (6)	0.055 (7)
C21A	0.040 (4)	0.062 (5)	0.070 (5)	0.006 (4)	−0.009 (3)	−0.018 (4)
C22A	0.057 (7)	0.111 (10)	0.166 (11)	0.032 (7)	−0.022 (7)	−0.077 (9)
C23A	0.045 (4)	0.065 (5)	0.065 (5)	0.003 (4)	0.016 (3)	−0.007 (4)
C15A	0.094 (8)	0.034 (4)	0.075 (6)	0.004 (5)	−0.016 (6)	−0.010 (4)
C16A	0.186 (15)	0.070 (8)	0.078 (8)	0.014 (9)	0.013 (8)	−0.021 (6)
C11A	0.124 (10)	0.028 (5)	0.105 (8)	−0.010 (6)	−0.020 (7)	−0.023 (5)
C12A	0.093 (9)	0.104 (10)	0.102 (9)	−0.036 (8)	−0.013 (7)	−0.033 (8)
C13A	0.070 (7)	0.048 (6)	0.071 (6)	0.002 (5)	−0.007 (5)	0.001 (5)
C14A	0.096 (9)	0.061 (7)	0.105 (9)	0.001 (7)	0.013 (7)	0.005 (7)
C17A	0.093 (9)	0.060 (7)	0.137 (11)	0.042 (7)	0.034 (8)	0.007 (7)
C18A	0.089 (10)	0.128 (13)	0.164 (14)	0.037 (9)	0.002 (9)	0.026 (10)
C24B	0.076 (7)	0.120 (11)	0.152 (11)	0.030 (8)	−0.033 (7)	−0.095 (10)
C24A	0.062 (7)	0.098 (9)	0.161 (11)	−0.018 (7)	0.027 (7)	−0.068 (8)
O63A	0.024 (2)	0.021 (2)	0.027 (2)	−0.0004 (17)	0.0073 (17)	0.0003 (16)
O63B	0.024 (2)	0.024 (2)	0.027 (2)	0.0006 (17)	0.0062 (18)	0.0040 (16)
O33A	0.025 (2)	0.023 (2)	0.039 (3)	0.0057 (19)	0.0019 (19)	0.0062 (19)
O84B	0.033 (2)	0.030 (2)	0.032 (2)	−0.003 (2)	0.0007 (18)	0.0052 (19)
O33B	0.027 (2)	0.025 (2)	0.031 (2)	−0.0007 (19)	0.0002 (18)	−0.0007 (17)
O82A	0.029 (3)	0.021 (2)	0.029 (3)	0.002 (2)	0.007 (2)	0.0037 (18)
O83A	0.027 (2)	0.022 (2)	0.034 (2)	−0.0004 (19)	0.0079 (17)	−0.0006 (18)
O83B	0.032 (2)	0.020 (2)	0.031 (2)	0.0044 (19)	0.0050 (17)	−0.0010 (18)
O72A	0.025 (2)	0.025 (2)	0.021 (2)	0.0009 (19)	0.0013 (18)	0.0002 (17)
O82B	0.028 (2)	0.020 (2)	0.026 (2)	0.0005 (19)	0.0077 (19)	0.0023 (16)
O21A	0.049 (3)	0.038 (3)	0.035 (3)	0.001 (2)	0.012 (2)	0.008 (2)
O74B	0.033 (2)	0.025 (2)	0.030 (2)	−0.0071 (19)	0.0081 (17)	−0.0047 (17)
O22A	0.046 (3)	0.039 (3)	0.059 (3)	−0.010 (2)	0.016 (2)	0.006 (2)
O42B	0.050 (3)	0.043 (3)	0.031 (2)	0.007 (2)	0.007 (2)	0.008 (2)
O73A	0.032 (2)	0.017 (2)	0.032 (2)	0.0013 (18)	0.0085 (17)	−0.0016 (17)
O72B	0.027 (2)	0.019 (2)	0.022 (2)	−0.0033 (19)	0.0052 (18)	−0.0002 (15)
O51A	0.049 (3)	0.041 (3)	0.035 (3)	−0.001 (2)	0.009 (2)	0.006 (2)
O73B	0.030 (2)	0.016 (2)	0.029 (2)	−0.0002 (18)	0.0079 (16)	−0.0002 (17)
O32B	0.041 (3)	0.038 (3)	0.034 (3)	−0.001 (2)	0.012 (2)	−0.006 (2)
O41A	0.044 (3)	0.035 (3)	0.053 (3)	0.012 (2)	0.014 (2)	0.009 (2)
O31B	0.045 (3)	0.044 (3)	0.045 (3)	0.009 (3)	0.010 (2)	0.010 (2)
O52A	0.049 (3)	0.039 (3)	0.059 (3)	−0.021 (2)	0.010 (2)	−0.003 (2)
O84A	0.032 (2)	0.028 (2)	0.037 (2)	−0.002 (2)	0.0071 (18)	0.0022 (19)
O13A	0.039 (3)	0.019 (2)	0.041 (2)	0.005 (2)	0.009 (2)	0.0049 (17)
O22B	0.047 (3)	0.044 (3)	0.055 (3)	−0.013 (2)	0.015 (2)	0.005 (2)
O43A	0.041 (3)	0.024 (2)	0.035 (2)	−0.001 (2)	0.0079 (19)	0.0022 (18)
O23A	0.032 (2)	0.030 (3)	0.036 (2)	−0.002 (2)	0.007 (2)	0.0015 (19)
O62B	0.049 (3)	0.041 (3)	0.039 (3)	−0.003 (3)	0.017 (2)	−0.006 (2)
O42A	0.041 (3)	0.047 (3)	0.030 (2)	0.002 (2)	0.0039 (19)	0.006 (2)
O81B	0.036 (3)	0.035 (3)	0.028 (2)	−0.003 (2)	0.0022 (19)	−0.0008 (19)
O11A	0.047 (3)	0.033 (3)	0.048 (3)	0.007 (2)	0.010 (2)	0.010 (2)
O61A	0.041 (3)	0.033 (3)	0.066 (3)	0.010 (2)	0.018 (2)	0.009 (2)
O62A	0.049 (3)	0.031 (3)	0.039 (3)	−0.006 (2)	0.013 (2)	−0.004 (2)
O12B	0.045 (3)	0.041 (3)	0.032 (2)	0.001 (2)	0.005 (2)	0.009 (2)
O52B	0.049 (3)	0.036 (3)	0.054 (3)	−0.012 (2)	0.011 (2)	0.004 (2)
O41B	0.050 (3)	0.028 (3)	0.052 (3)	0.016 (2)	0.008 (2)	0.007 (2)
O71A	0.039 (3)	0.041 (3)	0.029 (2)	0.000 (2)	0.006 (2)	−0.006 (2)
O71B	0.035 (3)	0.034 (3)	0.030 (2)	0.001 (2)	0.006 (2)	−0.0023 (19)
O32A	0.048 (3)	0.040 (3)	0.033 (3)	0.003 (3)	0.015 (2)	−0.002 (2)
O51B	0.056 (3)	0.042 (3)	0.034 (3)	0.005 (3)	0.011 (2)	0.006 (2)
O53A	0.024 (2)	0.030 (2)	0.038 (3)	0.0036 (19)	0.0071 (19)	−0.0018 (18)
O74A	0.036 (2)	0.027 (2)	0.029 (2)	−0.009 (2)	0.0059 (17)	−0.0055 (18)
O23B	0.031 (2)	0.031 (2)	0.035 (2)	−0.009 (2)	0.0106 (19)	−0.0003 (19)
O12A	0.049 (3)	0.043 (3)	0.030 (2)	0.003 (2)	0.006 (2)	0.011 (2)
O53B	0.026 (2)	0.032 (2)	0.039 (3)	0.0034 (19)	0.0110 (19)	0.0025 (19)
O61B	0.038 (3)	0.040 (3)	0.064 (3)	0.014 (2)	0.016 (2)	0.004 (2)
O81A	0.042 (3)	0.036 (3)	0.031 (2)	−0.004 (2)	0.005 (2)	0.002 (2)
O31A	0.048 (3)	0.041 (3)	0.054 (3)	0.013 (3)	0.018 (2)	0.015 (2)
O11B	0.043 (3)	0.038 (3)	0.052 (3)	0.018 (2)	0.004 (2)	0.006 (2)
O21B	0.046 (3)	0.037 (3)	0.032 (2)	−0.005 (2)	0.011 (2)	0.006 (2)
O13B	0.042 (3)	0.020 (2)	0.041 (3)	−0.002 (2)	0.007 (2)	0.0065 (18)
O43B	0.041 (3)	0.027 (2)	0.036 (2)	0.002 (2)	0.012 (2)	0.0104 (18)
Mo8B	0.0270 (3)	0.0207 (3)	0.0225 (3)	−0.0004 (2)	0.0030 (2)	−0.0018 (2)
Mo6B	0.0306 (3)	0.0261 (3)	0.0298 (3)	0.0032 (2)	0.0105 (2)	0.0000 (2)
Mo8A	0.0264 (3)	0.0210 (3)	0.0224 (3)	−0.0009 (2)	0.0030 (2)	−0.0005 (2)
Mo7A	0.0288 (3)	0.0222 (3)	0.0226 (3)	−0.0008 (2)	0.0030 (2)	−0.0018 (2)
Mo7B	0.0260 (3)	0.0204 (2)	0.0226 (3)	−0.0012 (2)	0.0030 (2)	−0.0008 (2)
Mo5B	0.0348 (3)	0.0273 (3)	0.0314 (3)	−0.0043 (3)	0.0104 (2)	0.0050 (2)
Mo3A	0.0317 (3)	0.0230 (3)	0.0318 (3)	0.0043 (2)	0.0112 (2)	0.0006 (2)
Mo3B	0.0278 (3)	0.0247 (3)	0.0291 (3)	0.0021 (2)	0.0088 (2)	0.0007 (2)
Mo2A	0.0308 (3)	0.0250 (3)	0.0317 (3)	−0.0031 (2)	0.0088 (2)	0.0037 (2)
Mo2B	0.0332 (3)	0.0248 (3)	0.0311 (3)	−0.0058 (2)	0.0087 (2)	0.0031 (2)
Mo6A	0.0297 (3)	0.0225 (3)	0.0304 (3)	0.0024 (2)	0.0108 (2)	0.0003 (2)
Mo4A	0.0326 (3)	0.0226 (3)	0.0297 (3)	0.0047 (2)	0.0060 (2)	0.0066 (2)
Mo5A	0.0335 (3)	0.0238 (3)	0.0319 (3)	−0.0051 (2)	0.0095 (2)	0.0009 (2)
Mo4B	0.0321 (3)	0.0242 (3)	0.0276 (3)	0.0052 (2)	0.0049 (2)	0.0056 (2)
Mo1A	0.0307 (3)	0.0237 (3)	0.0288 (3)	0.0043 (2)	0.0054 (2)	0.0070 (2)
Mo1B	0.0312 (3)	0.0230 (3)	0.0283 (3)	0.0049 (2)	0.0042 (2)	0.0055 (2)
N1B	0.048 (4)	0.024 (3)	0.061 (4)	0.002 (3)	−0.004 (3)	−0.011 (3)
N3A	0.054 (4)	0.024 (3)	0.053 (4)	0.001 (3)	−0.004 (3)	−0.010 (3)
N3B	0.071 (6)	0.028 (4)	0.075 (5)	0.005 (4)	−0.007 (4)	−0.013 (4)
N2B	0.028 (2)	0.044 (3)	0.043 (3)	0.0034 (19)	0.0096 (18)	−0.003 (2)
N2A	0.026 (2)	0.049 (3)	0.044 (3)	0.004 (2)	0.0108 (19)	0.000 (2)
N1A	0.064 (5)	0.031 (4)	0.079 (5)	0.016 (4)	−0.016 (4)	−0.020 (4)
N4A	0.048 (3)	0.041 (3)	0.021 (2)	−0.002 (2)	0.0058 (18)	−0.0018 (18)
N4B	0.049 (3)	0.036 (3)	0.025 (2)	0.001 (2)	0.0092 (19)	0.0042 (18)

### Geometric parameters (Å, °)

**Table:**

C11B—C12B	1.484 (15)	C15A—H15C	0.9700
C11B—N1B	1.506 (12)	C15A—H15D	0.9700
C11B—H11F	0.9700	C16A—H16C	0.9600
C11B—H11G	0.9700	C16A—H16E	0.9600
C12B—H12F	0.9600	C16A—H16D	0.9600
C12B—H12H	0.9600	C11A—C12A	1.479 (17)
C12B—H12G	0.9600	C11A—N1A	1.534 (14)
C17B—N1B	1.494 (12)	C11A—H11C	0.9700
C17B—C18B	1.518 (16)	C11A—H11D	0.9700
C17B—H17G	0.9700	C12A—H12C	0.9600
C17B—H17F	0.9700	C12A—H12E	0.9600
C18B—H18H	0.9600	C12A—H12D	0.9600
C18B—H18F	0.9600	C13A—N1A	1.505 (13)
C18B—H18G	0.9600	C13A—C14A	1.527 (15)
C13B—C14B	1.506 (13)	C13A—H13D	0.9700
C13B—N1B	1.512 (11)	C13A—H13C	0.9700
C13B—H13G	0.9700	C14A—H14E	0.9600
C13B—H13F	0.9700	C14A—H14C	0.9600
C14B—H14H	0.9600	C14A—H14D	0.9600
C14B—H14F	0.9600	C17A—N1A	1.485 (14)
C14B—H14G	0.9600	C17A—C18A	1.513 (19)
C15B—C16B	1.494 (13)	C17A—H17D	0.9700
C15B—N1B	1.506 (11)	C17A—H17C	0.9700
C15B—H15F	0.9700	C18A—H18E	0.9600
C15B—H15G	0.9700	C18A—H18C	0.9600
C16B—H16F	0.9600	C18A—H18D	0.9600
C16B—H16H	0.9600	C24B—H24F	0.9600
C16B—H16G	0.9600	C24B—H24H	0.9600
C37A—C38A	1.508 (12)	C24B—H24G	0.9600
C37A—N3A	1.518 (10)	C24A—H24C	0.9600
C37A—H37C	0.9700	C24A—H24D	0.9600
C37A—H37D	0.9700	C24A—H24E	0.9600
C38A—H38C	0.9600	O63A—Mo7A	1.944 (5)
C38A—H38D	0.9600	O63A—Mo1A	2.010 (4)
C38A—H38E	0.9600	O63A—Mo6A	2.310 (4)
C33A—C34A	1.489 (14)	O63B—Mo7B	1.943 (5)
C33A—N3A	1.519 (12)	O63B—Mo1B	2.005 (5)
C33A—H33C	0.9700	O63B—Mo6B	2.320 (4)
C33A—H33D	0.9700	O33A—Mo8A	1.946 (5)
C34A—H34E	0.9600	O33A—Mo4A	2.010 (5)
C34A—H34D	0.9600	O33A—Mo3A	2.336 (5)
C34A—H34C	0.9600	O84B—Mo8B	1.749 (5)
C31A—C32A	1.522 (13)	O84B—Mo2B	2.246 (4)
C31A—N3A	1.529 (11)	O33B—Mo8B	1.952 (5)
C31A—H31C	0.9700	O33B—Mo4B	2.005 (5)
C31A—H31D	0.9700	O33B—Mo3B	2.334 (5)
C32A—H32C	0.9600	O82A—Mo8A	2.130 (5)
C32A—H32D	0.9600	O82A—Mo4A	2.326 (5)
C32A—H32E	0.9600	O82A—Mo6A	2.349 (5)
C35A—N3A	1.515 (11)	O82A—Mo7A	2.353 (5)
C35A—C36A	1.528 (14)	O82A—Mo5A	2.478 (5)
C35A—H35C	0.9700	O83A—Mo8A	1.951 (5)
C35A—H35D	0.9700	O83A—Mo6A	2.011 (5)
C36A—H36D	0.9600	O83A—Mo1A	2.309 (4)
C36A—H36E	0.9600	O83B—Mo8B	1.958 (5)
C36A—H36C	0.9600	O83B—Mo6B	1.995 (5)
C31B—C32B	1.489 (14)	O83B—Mo1B	2.340 (4)
C31B—N3B	1.550 (13)	O72A—Mo7A	2.142 (5)
C31B—H31F	0.9700	O72A—Mo1A	2.312 (5)
C31B—H31G	0.9700	O72A—Mo3A	2.350 (5)
C32B—H32F	0.9600	O72A—Mo8A	2.363 (4)
C32B—H32G	0.9600	O72A—Mo2A	2.478 (5)
C32B—H32H	0.9600	O82B—Mo8B	2.108 (5)
C36B—C35B	1.470 (15)	O82B—Mo4B	2.338 (5)
C36B—H36G	0.9600	O82B—Mo7B	2.347 (5)
C36B—H36H	0.9600	O82B—Mo6B	2.385 (5)
C36B—H36F	0.9600	O82B—Mo5B	2.454 (5)
C35B—N3B	1.514 (13)	O21A—Mo2A	1.719 (5)
C35B—H35F	0.9700	O74B—Mo7B	1.759 (5)
C35B—H35G	0.9700	O74B—Mo5B	2.240 (4)
C33B—C34B	1.474 (16)	O22A—Mo2A	1.692 (5)
C33B—N3B	1.506 (14)	O42B—Mo4B	1.702 (5)
C33B—H33F	0.9700	O73A—Mo7A	1.944 (5)
C33B—H33G	0.9700	O73A—Mo3A	2.004 (5)
C34B—H34H	0.9600	O73A—Mo4A	2.314 (4)
C34B—H34G	0.9600	O72B—Mo7B	2.125 (5)
C34B—H34F	0.9600	O72B—Mo1B	2.338 (5)
C38B—C37B	1.520 (14)	O72B—Mo8B	2.359 (4)
C38B—H38F	0.9600	O72B—Mo3B	2.384 (5)
C38B—H38G	0.9600	O72B—Mo2B	2.426 (5)
C38B—H38H	0.9600	O51A—Mo5A	1.704 (5)
C37B—N3B	1.502 (11)	O73B—Mo7B	1.953 (5)
C37B—H37G	0.9700	O73B—Mo3B	2.003 (5)
C37B—H37F	0.9700	O73B—Mo4B	2.325 (4)
C26B—C25B	1.492 (12)	O32B—Mo3B	1.697 (5)
C26B—H26H	0.9600	O41A—Mo4A	1.691 (5)
C26B—H26F	0.9600	O31B—Mo3B	1.688 (5)
C26B—H26G	0.9600	O52A—Mo5A	1.700 (5)
C25B—N2B	1.544 (10)	O84A—Mo8A	1.746 (5)
C25B—H25G	0.9700	O84A—Mo2A	2.239 (4)
C25B—H25F	0.9700	O13A—Mo1A	1.894 (5)
C27B—N2B	1.498 (10)	O13A—Mo2A	1.919 (5)
C27B—C28B	1.522 (12)	O22B—Mo2B	1.697 (5)
C27B—H27F	0.9700	O43A—Mo4A	1.892 (5)
C27B—H27G	0.9700	O43A—Mo5A	1.929 (5)
C28B—H28G	0.9600	O23A—Mo3A	1.901 (5)
C28B—H28F	0.9600	O23A—Mo2A	1.915 (5)
C28B—H28H	0.9600	O62B—Mo6B	1.713 (5)
C21B—N2B	1.498 (10)	O42A—Mo4A	1.704 (5)
C21B—C22B	1.505 (13)	O81B—Mo8B	1.692 (5)
C21B—H21F	0.9700	O11A—Mo1A	1.697 (5)
C21B—H21G	0.9700	O61A—Mo6A	1.699 (5)
C22B—H22G	0.9600	O62A—Mo6A	1.712 (5)
C22B—H22H	0.9600	O12B—Mo1B	1.718 (5)
C22B—H22F	0.9600	O52B—Mo5B	1.691 (5)
C23B—C24B	1.513 (13)	O41B—Mo4B	1.686 (5)
C23B—N2B	1.521 (11)	O71A—Mo7A	1.700 (5)
C23B—H23F	0.9700	O71B—Mo7B	1.697 (5)
C23B—H23G	0.9700	O32A—Mo3A	1.698 (5)
C25A—N2A	1.524 (10)	O51B—Mo5B	1.705 (5)
C25A—C26A	1.529 (13)	O53A—Mo6A	1.887 (5)
C25A—H25D	0.9700	O53A—Mo5A	1.918 (5)
C25A—H25C	0.9700	O74A—Mo7A	1.741 (5)
C26A—H26E	0.9600	O74A—Mo5A	2.274 (4)
C26A—H26D	0.9600	O23B—Mo3B	1.896 (5)
C26A—H26C	0.9600	O23B—Mo2B	1.907 (5)
C27A—N2A	1.483 (10)	O12A—Mo1A	1.713 (5)
C27A—C28A	1.527 (12)	O53B—Mo6B	1.891 (5)
C27A—H27D	0.9700	O53B—Mo5B	1.922 (5)
C27A—H27C	0.9700	O61B—Mo6B	1.691 (5)
C28A—H28D	0.9600	O81A—Mo8A	1.690 (5)
C28A—H28E	0.9600	O31A—Mo3A	1.691 (5)
C28A—H28C	0.9600	O11B—Mo1B	1.707 (5)
C21A—C22A	1.484 (13)	O21B—Mo2B	1.716 (5)
C21A—N2A	1.512 (11)	O13B—Mo1B	1.877 (5)
C21A—H21C	0.9700	O13B—Mo2B	1.937 (5)
C21A—H21D	0.9700	O43B—Mo4B	1.882 (5)
C22A—H22D	0.9600	O43B—Mo5B	1.939 (5)
C22A—H22C	0.9600	N4A—H1N	0.82
C22A—H22E	0.9600	N4A—H2N	0.96
C23A—N2A	1.514 (10)	N4A—H3N	0.73
C23A—C24A	1.539 (13)	N4A—H4N	0.96
C23A—H23C	0.9700	N4B—H5N	0.85
C23A—H23D	0.9700	N4B—H6N	0.95
C15A—C16A	1.488 (15)	N4B—H7N	0.97
C15A—N1A	1.492 (13)	N4B—H8N	0.96
			
C12B—C11B—N1B	116.2 (8)	Mo8A—O72A—Mo2A	90.99 (16)
C12B—C11B—H11F	108.2	Mo8B—O82B—Mo4B	92.95 (19)
N1B—C11B—H11F	108.2	Mo8B—O82B—Mo7B	104.55 (18)
C12B—C11B—H11G	108.2	Mo4B—O82B—Mo7B	97.53 (19)
N1B—C11B—H11G	108.2	Mo8B—O82B—Mo6B	92.2 (2)
H11F—C11B—H11G	107.4	Mo4B—O82B—Mo6B	163.2 (2)
C11B—C12B—H12F	109.5	Mo7B—O82B—Mo6B	96.67 (18)
C11B—C12B—H12H	109.5	Mo8B—O82B—Mo5B	164.2 (2)
H12F—C12B—H12H	109.5	Mo4B—O82B—Mo5B	85.87 (17)
C11B—C12B—H12G	109.5	Mo7B—O82B—Mo5B	91.23 (17)
H12F—C12B—H12G	109.5	Mo6B—O82B—Mo5B	84.84 (15)
H12H—C12B—H12G	109.5	Mo7B—O74B—Mo5B	117.8 (2)
N1B—C17B—C18B	117.4 (8)	Mo7A—O73A—Mo3A	110.3 (2)
N1B—C17B—H17G	108.0	Mo7A—O73A—Mo4A	110.8 (2)
C18B—C17B—H17G	108.0	Mo3A—O73A—Mo4A	104.0 (2)
N1B—C17B—H17F	108.0	Mo7B—O72B—Mo1B	92.30 (18)
C18B—C17B—H17F	108.0	Mo7B—O72B—Mo8B	103.61 (17)
H17G—C17B—H17F	107.2	Mo1B—O72B—Mo8B	97.42 (18)
C17B—C18B—H18H	109.5	Mo7B—O72B—Mo3B	91.93 (19)
C17B—C18B—H18F	109.5	Mo1B—O72B—Mo3B	164.1 (2)
H18H—C18B—H18F	109.5	Mo8B—O72B—Mo3B	96.50 (17)
C17B—C18B—H18G	109.5	Mo7B—O72B—Mo2B	164.5 (2)
H18H—C18B—H18G	109.5	Mo1B—O72B—Mo2B	86.48 (17)
H18F—C18B—H18G	109.5	Mo8B—O72B—Mo2B	91.86 (16)
C14B—C13B—N1B	116.7 (9)	Mo3B—O72B—Mo2B	85.35 (15)
C14B—C13B—H13G	108.1	Mo7B—O73B—Mo3B	110.3 (2)
N1B—C13B—H13G	108.1	Mo7B—O73B—Mo4B	110.6 (2)
C14B—C13B—H13F	108.1	Mo3B—O73B—Mo4B	103.31 (19)
N1B—C13B—H13F	108.1	Mo8A—O84A—Mo2A	119.6 (2)
H13G—C13B—H13F	107.3	Mo1A—O13A—Mo2A	118.4 (3)
C13B—C14B—H14H	109.5	Mo4A—O43A—Mo5A	117.6 (3)
C13B—C14B—H14F	109.5	Mo3A—O23A—Mo2A	117.5 (3)
H14H—C14B—H14F	109.5	Mo6A—O53A—Mo5A	117.8 (2)
C13B—C14B—H14G	109.5	Mo7A—O74A—Mo5A	118.3 (2)
H14H—C14B—H14G	109.5	Mo3B—O23B—Mo2B	118.0 (3)
H14F—C14B—H14G	109.5	Mo6B—O53B—Mo5B	117.8 (3)
C16B—C15B—N1B	118.1 (8)	Mo1B—O13B—Mo2B	117.7 (3)
C16B—C15B—H15F	107.8	Mo4B—O43B—Mo5B	117.4 (3)
N1B—C15B—H15F	107.8	O81B—Mo8B—O84B	104.9 (2)
C16B—C15B—H15G	107.8	O81B—Mo8B—O33B	101.1 (2)
N1B—C15B—H15G	107.8	O84B—Mo8B—O33B	96.0 (2)
H15F—C15B—H15G	107.1	O81B—Mo8B—O83B	100.7 (2)
C15B—C16B—H16F	109.5	O84B—Mo8B—O83B	96.4 (2)
C15B—C16B—H16H	109.5	O33B—Mo8B—O83B	151.2 (2)
H16F—C16B—H16H	109.5	O81B—Mo8B—O82B	99.2 (2)
C15B—C16B—H16G	109.5	O84B—Mo8B—O82B	155.90 (19)
H16F—C16B—H16G	109.5	O33B—Mo8B—O82B	78.9 (2)
H16H—C16B—H16G	109.5	O83B—Mo8B—O82B	79.2 (2)
C38A—C37A—N3A	115.5 (7)	O81B—Mo8B—O72B	175.1 (2)
C38A—C37A—H37C	108.4	O84B—Mo8B—O72B	79.96 (19)
N3A—C37A—H37C	108.4	O33B—Mo8B—O72B	78.62 (19)
C38A—C37A—H37D	108.4	O83B—Mo8B—O72B	78.13 (18)
N3A—C37A—H37D	108.4	O82B—Mo8B—O72B	75.94 (17)
H37C—C37A—H37D	107.5	O61B—Mo6B—O62B	104.9 (3)
C37A—C38A—H38C	109.5	O61B—Mo6B—O53B	102.8 (2)
C37A—C38A—H38D	109.5	O62B—Mo6B—O53B	100.6 (3)
H38C—C38A—H38D	109.5	O61B—Mo6B—O83B	101.2 (2)
C37A—C38A—H38E	109.5	O62B—Mo6B—O83B	96.8 (2)
H38C—C38A—H38E	109.5	O53B—Mo6B—O83B	145.5 (2)
H38D—C38A—H38E	109.5	O61B—Mo6B—O63B	90.6 (2)
C34A—C33A—N3A	115.5 (8)	O62B—Mo6B—O63B	162.4 (2)
C34A—C33A—H33C	108.4	O53B—Mo6B—O63B	83.6 (2)
N3A—C33A—H33C	108.4	O83B—Mo6B—O63B	71.70 (18)
C34A—C33A—H33D	108.4	O61B—Mo6B—O82B	161.2 (2)
N3A—C33A—H33D	108.4	O62B—Mo6B—O82B	93.4 (2)
H33C—C33A—H33D	107.5	O53B—Mo6B—O82B	77.33 (19)
C33A—C34A—H34E	109.5	O83B—Mo6B—O82B	72.02 (19)
C33A—C34A—H34D	109.5	O63B—Mo6B—O82B	70.63 (16)
H34E—C34A—H34D	109.5	O81A—Mo8A—O84A	104.8 (2)
C33A—C34A—H34C	109.5	O81A—Mo8A—O33A	101.1 (2)
H34E—C34A—H34C	109.5	O84A—Mo8A—O33A	96.5 (2)
H34D—C34A—H34C	109.5	O81A—Mo8A—O83A	101.3 (2)
C32A—C31A—N3A	114.1 (8)	O84A—Mo8A—O83A	96.2 (2)
C32A—C31A—H31C	108.7	O33A—Mo8A—O83A	150.4 (2)
N3A—C31A—H31C	108.7	O81A—Mo8A—O82A	99.6 (2)
C32A—C31A—H31D	108.7	O84A—Mo8A—O82A	155.6 (2)
N3A—C31A—H31D	108.7	O33A—Mo8A—O82A	78.7 (2)
H31C—C31A—H31D	107.6	O83A—Mo8A—O82A	78.7 (2)
C31A—C32A—H32C	109.5	O81A—Mo8A—O72A	175.1 (2)
C31A—C32A—H32D	109.5	O84A—Mo8A—O72A	80.1 (2)
H32C—C32A—H32D	109.5	O33A—Mo8A—O72A	78.68 (19)
C31A—C32A—H32E	109.5	O83A—Mo8A—O72A	77.38 (18)
H32C—C32A—H32E	109.5	O82A—Mo8A—O72A	75.50 (17)
H32D—C32A—H32E	109.5	O71A—Mo7A—O74A	103.9 (2)
N3A—C35A—C36A	115.2 (8)	O71A—Mo7A—O63A	101.5 (2)
N3A—C35A—H35C	108.5	O74A—Mo7A—O63A	96.8 (2)
C36A—C35A—H35C	108.5	O71A—Mo7A—O73A	101.5 (2)
N3A—C35A—H35D	108.5	O74A—Mo7A—O73A	96.7 (2)
C36A—C35A—H35D	108.5	O63A—Mo7A—O73A	149.6 (2)
H35C—C35A—H35D	107.5	O71A—Mo7A—O72A	99.4 (2)
C35A—C36A—H36D	109.5	O74A—Mo7A—O72A	156.68 (18)
C35A—C36A—H36E	109.5	O63A—Mo7A—O72A	78.36 (19)
H36D—C36A—H36E	109.5	O73A—Mo7A—O72A	78.47 (19)
C35A—C36A—H36C	109.5	O71A—Mo7A—O82A	174.9 (2)
H36D—C36A—H36C	109.5	O74A—Mo7A—O82A	81.16 (19)
H36E—C36A—H36C	109.5	O63A—Mo7A—O82A	77.95 (19)
C32B—C31B—N3B	115.2 (10)	O73A—Mo7A—O82A	77.35 (19)
C32B—C31B—H31F	108.5	O72A—Mo7A—O82A	75.52 (16)
N3B—C31B—H31F	108.5	O71B—Mo7B—O74B	103.5 (2)
C32B—C31B—H31G	108.5	O71B—Mo7B—O63B	102.2 (2)
N3B—C31B—H31G	108.5	O74B—Mo7B—O63B	96.2 (2)
H31F—C31B—H31G	107.5	O71B—Mo7B—O73B	100.7 (2)
C31B—C32B—H32F	109.5	O74B—Mo7B—O73B	96.2 (2)
C31B—C32B—H32G	109.5	O63B—Mo7B—O73B	150.6 (2)
H32F—C32B—H32G	109.5	O71B—Mo7B—O72B	99.7 (2)
C31B—C32B—H32H	109.5	O74B—Mo7B—O72B	156.80 (18)
H32F—C32B—H32H	109.5	O63B—Mo7B—O72B	78.9 (2)
H32G—C32B—H32H	109.5	O73B—Mo7B—O72B	79.3 (2)
C35B—C36B—H36G	109.5	O71B—Mo7B—O82B	175.5 (2)
C35B—C36B—H36H	109.5	O74B—Mo7B—O82B	80.90 (19)
H36G—C36B—H36H	109.5	O63B—Mo7B—O82B	78.08 (19)
C35B—C36B—H36F	109.5	O73B—Mo7B—O82B	77.73 (19)
H36G—C36B—H36F	109.5	O72B—Mo7B—O82B	75.89 (16)
H36H—C36B—H36F	109.5	O52B—Mo5B—O51B	104.8 (3)
C36B—C35B—N3B	118.3 (10)	O52B—Mo5B—O53B	104.0 (2)
C36B—C35B—H35F	107.7	O51B—Mo5B—O53B	97.6 (3)
N3B—C35B—H35F	107.7	O52B—Mo5B—O43B	101.5 (2)
C36B—C35B—H35G	107.7	O51B—Mo5B—O43B	97.2 (2)
N3B—C35B—H35G	107.7	O53B—Mo5B—O43B	145.9 (2)
H35F—C35B—H35G	107.1	O52B—Mo5B—O74B	92.9 (2)
C34B—C33B—N3B	115.5 (10)	O51B—Mo5B—O74B	162.2 (2)
C34B—C33B—H33F	108.4	O53B—Mo5B—O74B	78.6 (2)
N3B—C33B—H33F	108.4	O43B—Mo5B—O74B	77.98 (19)
C34B—C33B—H33G	108.4	O52B—Mo5B—O82B	162.9 (2)
N3B—C33B—H33G	108.4	O51B—Mo5B—O82B	92.1 (2)
H33F—C33B—H33G	107.5	O53B—Mo5B—O82B	75.04 (19)
C33B—C34B—H34H	109.5	O43B—Mo5B—O82B	73.9 (2)
C33B—C34B—H34G	109.5	O74B—Mo5B—O82B	70.10 (17)
H34H—C34B—H34G	109.5	O31A—Mo3A—O32A	104.3 (3)
C33B—C34B—H34F	109.5	O31A—Mo3A—O23A	102.5 (2)
H34H—C34B—H34F	109.5	O32A—Mo3A—O23A	100.7 (2)
H34G—C34B—H34F	109.5	O31A—Mo3A—O73A	100.6 (2)
C37B—C38B—H38F	109.5	O32A—Mo3A—O73A	97.3 (2)
C37B—C38B—H38G	109.5	O23A—Mo3A—O73A	145.9 (2)
H38F—C38B—H38G	109.5	O31A—Mo3A—O33A	90.2 (2)
C37B—C38B—H38H	109.5	O32A—Mo3A—O33A	163.3 (2)
H38F—C38B—H38H	109.5	O23A—Mo3A—O33A	83.7 (2)
H38G—C38B—H38H	109.5	O73A—Mo3A—O33A	71.56 (18)
N3B—C37B—C38B	115.6 (9)	O31A—Mo3A—O72A	162.0 (2)
N3B—C37B—H37G	108.4	O32A—Mo3A—O72A	93.2 (2)
C38B—C37B—H37G	108.4	O23A—Mo3A—O72A	77.9 (2)
N3B—C37B—H37F	108.4	O73A—Mo3A—O72A	72.48 (19)
C38B—C37B—H37F	108.4	O33A—Mo3A—O72A	71.89 (15)
H37G—C37B—H37F	107.4	O31B—Mo3B—O32B	104.8 (3)
C25B—C26B—H26H	109.5	O31B—Mo3B—O23B	103.6 (2)
C25B—C26B—H26F	109.5	O32B—Mo3B—O23B	100.6 (2)
H26H—C26B—H26F	109.5	O31B—Mo3B—O73B	101.1 (2)
C25B—C26B—H26G	109.5	O32B—Mo3B—O73B	96.8 (2)
H26H—C26B—H26G	109.5	O23B—Mo3B—O73B	144.9 (2)
H26F—C26B—H26G	109.5	O31B—Mo3B—O33B	89.9 (2)
C26B—C25B—N2B	116.5 (7)	O32B—Mo3B—O33B	163.0 (2)
C26B—C25B—H25G	108.2	O23B—Mo3B—O33B	83.64 (19)
N2B—C25B—H25G	108.2	O73B—Mo3B—O33B	71.68 (17)
C26B—C25B—H25F	108.2	O31B—Mo3B—O72B	161.2 (2)
N2B—C25B—H25F	108.2	O32B—Mo3B—O72B	93.6 (2)
H25G—C25B—H25F	107.3	O23B—Mo3B—O72B	76.43 (19)
N2B—C27B—C28B	115.2 (7)	O73B—Mo3B—O72B	72.24 (18)
N2B—C27B—H27F	108.5	O33B—Mo3B—O72B	71.28 (16)
C28B—C27B—H27F	108.5	O22A—Mo2A—O21A	104.5 (3)
N2B—C27B—H27G	108.5	O22A—Mo2A—O23A	103.8 (2)
C28B—C27B—H27G	108.5	O21A—Mo2A—O23A	97.7 (2)
H27F—C27B—H27G	107.5	O22A—Mo2A—O13A	102.7 (2)
C27B—C28B—H28G	109.5	O21A—Mo2A—O13A	97.6 (2)
C27B—C28B—H28F	109.5	O23A—Mo2A—O13A	144.8 (2)
H28G—C28B—H28F	109.5	O22A—Mo2A—O84A	93.3 (2)
C27B—C28B—H28H	109.5	O21A—Mo2A—O84A	162.3 (2)
H28G—C28B—H28H	109.5	O23A—Mo2A—O84A	77.75 (19)
H28F—C28B—H28H	109.5	O13A—Mo2A—O84A	78.04 (18)
N2B—C21B—C22B	113.5 (7)	O22A—Mo2A—O72A	162.5 (2)
N2B—C21B—H21F	108.9	O21A—Mo2A—O72A	93.0 (2)
C22B—C21B—H21F	108.9	O23A—Mo2A—O72A	74.42 (19)
N2B—C21B—H21G	108.9	O13A—Mo2A—O72A	73.28 (19)
C22B—C21B—H21G	108.9	O84A—Mo2A—O72A	69.23 (16)
H21F—C21B—H21G	107.7	O22B—Mo2B—O21B	104.4 (3)
C21B—C22B—H22G	109.5	O22B—Mo2B—O23B	104.5 (2)
C21B—C22B—H22H	109.5	O21B—Mo2B—O23B	97.4 (2)
H22G—C22B—H22H	109.5	O22B—Mo2B—O13B	101.1 (2)
C21B—C22B—H22F	109.5	O21B—Mo2B—O13B	97.3 (2)
H22G—C22B—H22F	109.5	O23B—Mo2B—O13B	146.1 (2)
H22H—C22B—H22F	109.5	O22B—Mo2B—O84B	93.8 (2)
C24B—C23B—N2B	115.6 (7)	O21B—Mo2B—O84B	161.8 (2)
C24B—C23B—H23F	108.4	O23B—Mo2B—O84B	78.25 (19)
N2B—C23B—H23F	108.4	O13B—Mo2B—O84B	78.25 (19)
C24B—C23B—H23G	108.4	O22B—Mo2B—O72B	163.6 (2)
N2B—C23B—H23G	108.4	O21B—Mo2B—O72B	91.9 (2)
H23F—C23B—H23G	107.4	O23B—Mo2B—O72B	75.19 (19)
N2A—C25A—C26A	113.0 (7)	O13B—Mo2B—O72B	73.91 (19)
N2A—C25A—H25D	109.0	O84B—Mo2B—O72B	69.90 (17)
C26A—C25A—H25D	109.0	O61A—Mo6A—O62A	104.8 (3)
N2A—C25A—H25C	109.0	O61A—Mo6A—O53A	102.6 (2)
C26A—C25A—H25C	109.0	O62A—Mo6A—O53A	101.3 (2)
H25D—C25A—H25C	107.8	O61A—Mo6A—O83A	100.5 (2)
C25A—C26A—H26E	109.5	O62A—Mo6A—O83A	95.9 (2)
C25A—C26A—H26D	109.5	O53A—Mo6A—O83A	146.5 (2)
H26E—C26A—H26D	109.5	O61A—Mo6A—O63A	89.9 (2)
C25A—C26A—H26C	109.5	O62A—Mo6A—O63A	162.5 (2)
H26E—C26A—H26C	109.5	O53A—Mo6A—O63A	84.21 (19)
H26D—C26A—H26C	109.5	O83A—Mo6A—O63A	71.80 (17)
N2A—C27A—C28A	113.9 (7)	O61A—Mo6A—O82A	161.3 (2)
N2A—C27A—H27D	108.8	O62A—Mo6A—O82A	93.3 (2)
C28A—C27A—H27D	108.8	O53A—Mo6A—O82A	77.9 (2)
N2A—C27A—H27C	108.8	O83A—Mo6A—O82A	72.50 (19)
C28A—C27A—H27C	108.8	O63A—Mo6A—O82A	71.48 (16)
H27D—C27A—H27C	107.7	O41A—Mo4A—O42A	105.2 (3)
C27A—C28A—H28D	109.5	O41A—Mo4A—O43A	101.5 (2)
C27A—C28A—H28E	109.5	O42A—Mo4A—O43A	100.9 (2)
H28D—C28A—H28E	109.5	O41A—Mo4A—O33A	100.8 (2)
C27A—C28A—H28C	109.5	O42A—Mo4A—O33A	95.7 (2)
H28D—C28A—H28C	109.5	O43A—Mo4A—O33A	147.6 (2)
H28E—C28A—H28C	109.5	O41A—Mo4A—O73A	89.0 (2)
C22A—C21A—N2A	115.3 (7)	O42A—Mo4A—O73A	162.9 (2)
C22A—C21A—H21C	108.5	O43A—Mo4A—O73A	85.17 (18)
N2A—C21A—H21C	108.5	O33A—Mo4A—O73A	71.93 (18)
C22A—C21A—H21D	108.5	O41A—Mo4A—O82A	160.3 (2)
N2A—C21A—H21D	108.5	O42A—Mo4A—O82A	94.1 (2)
H21C—C21A—H21D	107.5	O43A—Mo4A—O82A	78.2 (2)
C21A—C22A—H22D	109.5	O33A—Mo4A—O82A	72.90 (19)
C21A—C22A—H22C	109.5	O73A—Mo4A—O82A	71.28 (17)
H22D—C22A—H22C	109.5	O52A—Mo5A—O51A	103.6 (3)
C21A—C22A—H22E	109.5	O52A—Mo5A—O53A	104.0 (2)
H22D—C22A—H22E	109.5	O51A—Mo5A—O53A	98.3 (3)
H22C—C22A—H22E	109.5	O52A—Mo5A—O43A	102.3 (3)
N2A—C23A—C24A	116.1 (7)	O51A—Mo5A—O43A	97.8 (2)
N2A—C23A—H23C	108.3	O53A—Mo5A—O43A	144.8 (2)
C24A—C23A—H23C	108.3	O52A—Mo5A—O74A	93.7 (2)
N2A—C23A—H23D	108.3	O51A—Mo5A—O74A	162.7 (2)
C24A—C23A—H23D	108.3	O53A—Mo5A—O74A	77.6 (2)
H23C—C23A—H23D	107.4	O43A—Mo5A—O74A	77.75 (19)
C16A—C15A—N1A	117.9 (9)	O52A—Mo5A—O82A	162.9 (2)
C16A—C15A—H15C	107.8	O51A—Mo5A—O82A	93.5 (2)
N1A—C15A—H15C	107.8	O53A—Mo5A—O82A	74.16 (19)
C16A—C15A—H15D	107.8	O43A—Mo5A—O82A	73.80 (19)
N1A—C15A—H15D	107.8	O74A—Mo5A—O82A	69.19 (17)
H15C—C15A—H15D	107.2	O41B—Mo4B—O42B	105.0 (3)
C15A—C16A—H16C	109.5	O41B—Mo4B—O43B	101.6 (2)
C15A—C16A—H16E	109.5	O42B—Mo4B—O43B	101.9 (2)
H16C—C16A—H16E	109.5	O41B—Mo4B—O33B	100.9 (2)
C15A—C16A—H16D	109.5	O42B—Mo4B—O33B	96.2 (2)
H16C—C16A—H16D	109.5	O43B—Mo4B—O33B	146.3 (2)
H16E—C16A—H16D	109.5	O41B—Mo4B—O73B	88.6 (2)
C12A—C11A—N1A	116.7 (9)	O42B—Mo4B—O73B	163.6 (2)
C12A—C11A—H11C	108.1	O43B—Mo4B—O73B	83.92 (18)
N1A—C11A—H11C	108.1	O33B—Mo4B—O73B	71.83 (18)
C12A—C11A—H11D	108.1	O41B—Mo4B—O82B	159.8 (2)
N1A—C11A—H11D	108.1	O42B—Mo4B—O82B	94.8 (2)
H11C—C11A—H11D	107.3	O43B—Mo4B—O82B	77.8 (2)
C11A—C12A—H12C	109.5	O33B—Mo4B—O82B	72.50 (19)
C11A—C12A—H12E	109.5	O73B—Mo4B—O82B	71.24 (17)
H12C—C12A—H12E	109.5	O11A—Mo1A—O12A	104.1 (3)
C11A—C12A—H12D	109.5	O11A—Mo1A—O13A	101.9 (2)
H12C—C12A—H12D	109.5	O12A—Mo1A—O13A	100.9 (2)
H12E—C12A—H12D	109.5	O11A—Mo1A—O63A	100.7 (2)
N1A—C13A—C14A	116.4 (10)	O12A—Mo1A—O63A	96.3 (2)
N1A—C13A—H13D	108.2	O13A—Mo1A—O63A	147.19 (19)
C14A—C13A—H13D	108.2	O11A—Mo1A—O83A	88.9 (2)
N1A—C13A—H13C	108.2	O12A—Mo1A—O83A	164.0 (2)
C14A—C13A—H13C	108.2	O13A—Mo1A—O83A	85.05 (17)
H13D—C13A—H13C	107.3	O63A—Mo1A—O83A	71.83 (18)
C13A—C14A—H14E	109.5	O11A—Mo1A—O72A	160.9 (2)
C13A—C14A—H14C	109.5	O12A—Mo1A—O72A	94.6 (2)
H14E—C14A—H14C	109.5	O13A—Mo1A—O72A	77.85 (19)
C13A—C14A—H14D	109.5	O63A—Mo1A—O72A	73.11 (18)
H14E—C14A—H14D	109.5	O83A—Mo1A—O72A	72.01 (16)
H14C—C14A—H14D	109.5	O11B—Mo1B—O12B	104.1 (3)
N1A—C17A—C18A	115.2 (12)	O11B—Mo1B—O13B	101.7 (2)
N1A—C17A—H17D	108.5	O12B—Mo1B—O13B	101.7 (2)
C18A—C17A—H17D	108.5	O11B—Mo1B—O63B	101.6 (2)
N1A—C17A—H17C	108.5	O12B—Mo1B—O63B	96.7 (2)
C18A—C17A—H17C	108.5	O13B—Mo1B—O63B	145.7 (2)
H17D—C17A—H17C	107.5	O11B—Mo1B—O72B	161.0 (2)
C17A—C18A—H18E	109.5	O12B—Mo1B—O72B	94.7 (2)
C17A—C18A—H18C	109.5	O13B—Mo1B—O72B	77.12 (19)
H18E—C18A—H18C	109.5	O63B—Mo1B—O72B	72.69 (18)
C17A—C18A—H18D	109.5	O11B—Mo1B—O83B	89.3 (2)
H18E—C18A—H18D	109.5	O12B—Mo1B—O83B	163.6 (2)
H18C—C18A—H18D	109.5	O13B—Mo1B—O83B	84.36 (19)
C23B—C24B—H24F	109.5	O63B—Mo1B—O83B	71.08 (19)
C23B—C24B—H24H	109.5	O72B—Mo1B—O83B	71.64 (16)
H24F—C24B—H24H	109.5	C17B—N1B—C15B	111.4 (8)
C23B—C24B—H24G	109.5	C17B—N1B—C11B	104.8 (7)
H24F—C24B—H24G	109.5	C15B—N1B—C11B	111.5 (8)
H24H—C24B—H24G	109.5	C17B—N1B—C13B	111.5 (7)
C23A—C24A—H24C	109.5	C15B—N1B—C13B	106.3 (7)
C23A—C24A—H24D	109.5	C11B—N1B—C13B	111.5 (8)
H24C—C24A—H24D	109.5	C35A—N3A—C37A	112.5 (7)
C23A—C24A—H24E	109.5	C35A—N3A—C33A	105.0 (7)
H24C—C24A—H24E	109.5	C37A—N3A—C33A	111.3 (7)
H24D—C24A—H24E	109.5	C35A—N3A—C31A	110.1 (7)
Mo7A—O63A—Mo1A	109.4 (2)	C37A—N3A—C31A	106.3 (6)
Mo7A—O63A—Mo6A	110.9 (2)	C33A—N3A—C31A	111.8 (8)
Mo1A—O63A—Mo6A	103.5 (2)	C37B—N3B—C33B	110.7 (8)
Mo7B—O63B—Mo1B	109.4 (2)	C37B—N3B—C35B	111.9 (9)
Mo7B—O63B—Mo6B	111.7 (2)	C33B—N3B—C35B	106.5 (8)
Mo1B—O63B—Mo6B	103.7 (2)	C37B—N3B—C31B	103.9 (7)
Mo8A—O33A—Mo4A	109.4 (2)	C33B—N3B—C31B	113.4 (8)
Mo8A—O33A—Mo3A	110.1 (2)	C35B—N3B—C31B	110.4 (9)
Mo4A—O33A—Mo3A	103.0 (2)	C21B—N2B—C27B	113.1 (7)
Mo8B—O84B—Mo2B	118.3 (2)	C21B—N2B—C23B	105.9 (6)
Mo8B—O33B—Mo4B	109.3 (2)	C27B—N2B—C23B	111.8 (7)
Mo8B—O33B—Mo3B	111.0 (2)	C21B—N2B—C25B	109.5 (6)
Mo4B—O33B—Mo3B	102.9 (2)	C27B—N2B—C25B	106.6 (6)
Mo8A—O82A—Mo4A	92.75 (19)	C23B—N2B—C25B	109.9 (7)
Mo8A—O82A—Mo6A	92.6 (2)	C27A—N2A—C21A	112.8 (7)
Mo4A—O82A—Mo6A	163.4 (2)	C27A—N2A—C23A	110.4 (6)
Mo8A—O82A—Mo7A	104.85 (18)	C21A—N2A—C23A	105.5 (6)
Mo4A—O82A—Mo7A	97.27 (19)	C27A—N2A—C25A	106.0 (6)
Mo6A—O82A—Mo7A	96.56 (18)	C21A—N2A—C25A	110.9 (7)
Mo8A—O82A—Mo5A	163.8 (2)	C23A—N2A—C25A	111.4 (7)
Mo4A—O82A—Mo5A	85.64 (17)	C17A—N1A—C15A	111.1 (9)
Mo6A—O82A—Mo5A	84.87 (16)	C17A—N1A—C13A	114.9 (9)
Mo7A—O82A—Mo5A	91.37 (18)	C15A—N1A—C13A	104.8 (8)
Mo8A—O83A—Mo6A	109.7 (2)	C17A—N1A—C11A	104.3 (9)
Mo8A—O83A—Mo1A	110.5 (2)	C15A—N1A—C11A	111.2 (9)
Mo6A—O83A—Mo1A	103.5 (2)	C13A—N1A—C11A	110.7 (9)
Mo8B—O83B—Mo6B	110.2 (2)	H1N—N4A—H2N	112
Mo8B—O83B—Mo1B	110.1 (2)	H1N—N4A—H3N	100
Mo6B—O83B—Mo1B	103.3 (2)	H2N—N4A—H3N	104
Mo7A—O72A—Mo1A	92.79 (17)	H1N—N4A—H4N	117
Mo7A—O72A—Mo3A	92.2 (2)	H2N—N4A—H4N	116
Mo1A—O72A—Mo3A	163.9 (2)	H3N—N4A—H4N	106
Mo7A—O72A—Mo8A	104.12 (18)	H5N—N4B—H6N	105
Mo1A—O72A—Mo8A	97.16 (18)	H5N—N4B—H7N	117
Mo3A—O72A—Mo8A	96.48 (17)	H6N—N4B—H7N	102
Mo7A—O72A—Mo2A	164.9 (2)	H5N—N4B—H8N	101
Mo1A—O72A—Mo2A	86.17 (17)	H6N—N4B—H8N	117
Mo3A—O72A—Mo2A	85.00 (15)	H7N—N4B—H8N	115
			
Mo2B—O84B—Mo8B—O81B	−179.8 (3)	Mo8B—O72B—Mo3B—O32B	177.3 (2)
Mo2B—O84B—Mo8B—O33B	−76.7 (3)	Mo2B—O72B—Mo3B—O32B	85.9 (2)
Mo2B—O84B—Mo8B—O83B	77.3 (3)	Mo7B—O72B—Mo3B—O23B	−178.8 (2)
Mo2B—O84B—Mo8B—O82B	−0.6 (7)	Mo1B—O72B—Mo3B—O23B	−73.5 (9)
Mo2B—O84B—Mo8B—O72B	0.6 (2)	Mo8B—O72B—Mo3B—O23B	77.2 (2)
Mo4B—O33B—Mo8B—O81B	−75.5 (3)	Mo2B—O72B—Mo3B—O23B	−14.12 (17)
Mo3B—O33B—Mo8B—O81B	171.6 (2)	Mo7B—O72B—Mo3B—O73B	17.25 (16)
Mo4B—O33B—Mo8B—O84B	178.0 (2)	Mo1B—O72B—Mo3B—O73B	122.6 (9)
Mo3B—O33B—Mo8B—O84B	65.1 (2)	Mo8B—O72B—Mo3B—O73B	−86.68 (18)
Mo4B—O33B—Mo8B—O83B	62.8 (5)	Mo2B—O72B—Mo3B—O73B	−178.03 (19)
Mo3B—O33B—Mo8B—O83B	−50.0 (5)	Mo7B—O72B—Mo3B—O33B	93.37 (17)
Mo4B—O33B—Mo8B—O82B	21.8 (2)	Mo1B—O72B—Mo3B—O33B	−161.3 (9)
Mo3B—O33B—Mo8B—O82B	−91.0 (2)	Mo8B—O72B—Mo3B—O33B	−10.57 (15)
Mo4B—O33B—Mo8B—O72B	99.5 (2)	Mo2B—O72B—Mo3B—O33B	−101.92 (17)
Mo3B—O33B—Mo8B—O72B	−13.32 (19)	Mo3A—O23A—Mo2A—O22A	177.8 (3)
Mo6B—O83B—Mo8B—O81B	75.3 (3)	Mo3A—O23A—Mo2A—O21A	70.7 (3)
Mo1B—O83B—Mo8B—O81B	−171.5 (2)	Mo3A—O23A—Mo2A—O13A	−44.3 (5)
Mo6B—O83B—Mo8B—O84B	−178.2 (2)	Mo3A—O23A—Mo2A—O84A	−91.8 (3)
Mo1B—O83B—Mo8B—O84B	−64.9 (3)	Mo3A—O23A—Mo2A—O72A	−20.3 (2)
Mo6B—O83B—Mo8B—O33B	−63.2 (5)	Mo1A—O13A—Mo2A—O22A	−179.1 (3)
Mo1B—O83B—Mo8B—O33B	50.1 (5)	Mo1A—O13A—Mo2A—O21A	−72.3 (3)
Mo6B—O83B—Mo8B—O82B	−22.2 (2)	Mo1A—O13A—Mo2A—O23A	42.8 (5)
Mo1B—O83B—Mo8B—O82B	91.1 (2)	Mo1A—O13A—Mo2A—O84A	90.2 (3)
Mo6B—O83B—Mo8B—O72B	−99.9 (2)	Mo1A—O13A—Mo2A—O72A	18.6 (2)
Mo1B—O83B—Mo8B—O72B	13.35 (19)	Mo8A—O84A—Mo2A—O22A	−179.7 (3)
Mo4B—O82B—Mo8B—O81B	82.0 (2)	Mo8A—O84A—Mo2A—O21A	0.1 (9)
Mo7B—O82B—Mo8B—O81B	−179.4 (2)	Mo8A—O84A—Mo2A—O23A	76.9 (3)
Mo6B—O82B—Mo8B—O81B	−82.0 (2)	Mo8A—O84A—Mo2A—O13A	−77.3 (3)
Mo5B—O82B—Mo8B—O81B	−3.3 (10)	Mo8A—O84A—Mo2A—O72A	−0.9 (2)
Mo4B—O82B—Mo8B—O84B	−97.2 (5)	Mo7A—O72A—Mo2A—O22A	−179.0 (8)
Mo7B—O82B—Mo8B—O84B	1.3 (6)	Mo1A—O72A—Mo2A—O22A	−92.5 (7)
Mo6B—O82B—Mo8B—O84B	98.8 (5)	Mo3A—O72A—Mo2A—O22A	101.0 (7)
Mo5B—O82B—Mo8B—O84B	177.5 (7)	Mo8A—O72A—Mo2A—O22A	4.6 (8)
Mo4B—O82B—Mo8B—O33B	−17.54 (16)	Mo7A—O72A—Mo2A—O21A	−2.8 (10)
Mo7B—O82B—Mo8B—O33B	81.0 (2)	Mo1A—O72A—Mo2A—O21A	83.7 (2)
Mo6B—O82B—Mo8B—O33B	178.5 (2)	Mo3A—O72A—Mo2A—O21A	−82.7 (2)
Mo5B—O82B—Mo8B—O33B	−102.8 (10)	Mo8A—O72A—Mo2A—O21A	−179.1 (2)
Mo4B—O82B—Mo8B—O83B	−178.8 (2)	Mo7A—O72A—Mo2A—O23A	94.4 (9)
Mo7B—O82B—Mo8B—O83B	−80.2 (2)	Mo1A—O72A—Mo2A—O23A	−179.1 (2)
Mo6B—O82B—Mo8B—O83B	17.26 (16)	Mo3A—O72A—Mo2A—O23A	14.44 (18)
Mo5B—O82B—Mo8B—O83B	95.9 (10)	Mo8A—O72A—Mo2A—O23A	−82.0 (2)
Mo4B—O82B—Mo8B—O72B	−98.45 (16)	Mo7A—O72A—Mo2A—O13A	−99.8 (10)
Mo7B—O82B—Mo8B—O72B	0.1 (2)	Mo1A—O72A—Mo2A—O13A	−13.29 (16)
Mo6B—O82B—Mo8B—O72B	97.58 (15)	Mo3A—O72A—Mo2A—O13A	−179.8 (2)
Mo5B—O82B—Mo8B—O72B	176.3 (9)	Mo8A—O72A—Mo2A—O13A	83.82 (19)
Mo7B—O72B—Mo8B—O81B	5(3)	Mo7A—O72A—Mo2A—O84A	176.9 (10)
Mo1B—O72B—Mo8B—O81B	−89 (3)	Mo1A—O72A—Mo2A—O84A	−96.54 (17)
Mo3B—O72B—Mo8B—O81B	99 (3)	Mo3A—O72A—Mo2A—O84A	96.98 (17)
Mo2B—O72B—Mo8B—O81B	−176 (3)	Mo8A—O72A—Mo2A—O84A	0.57 (15)
Mo7B—O72B—Mo8B—O84B	−179.6 (3)	Mo3B—O23B—Mo2B—O22B	176.7 (3)
Mo1B—O72B—Mo8B—O84B	86.2 (2)	Mo3B—O23B—Mo2B—O21B	69.7 (3)
Mo3B—O72B—Mo8B—O84B	−86.0 (2)	Mo3B—O23B—Mo2B—O13B	−45.3 (5)
Mo2B—O72B—Mo8B—O84B	−0.52 (19)	Mo3B—O23B—Mo2B—O84B	−92.3 (3)
Mo7B—O72B—Mo8B—O33B	−81.4 (2)	Mo3B—O23B—Mo2B—O72B	−20.3 (2)
Mo1B—O72B—Mo8B—O33B	−175.5 (2)	Mo1B—O13B—Mo2B—O22B	−176.6 (3)
Mo3B—O72B—Mo8B—O33B	12.23 (18)	Mo1B—O13B—Mo2B—O21B	−70.3 (3)
Mo2B—O72B—Mo8B—O33B	97.76 (19)	Mo1B—O13B—Mo2B—O23B	44.8 (5)
Mo7B—O72B—Mo8B—O83B	81.6 (2)	Mo1B—O13B—Mo2B—O84B	91.8 (3)
Mo1B—O72B—Mo8B—O83B	−12.64 (18)	Mo1B—O13B—Mo2B—O72B	19.6 (2)
Mo3B—O72B—Mo8B—O83B	175.1 (2)	Mo8B—O84B—Mo2B—O22B	−178.2 (3)
Mo2B—O72B—Mo8B—O83B	−99.3 (2)	Mo8B—O84B—Mo2B—O21B	−0.1 (8)
Mo7B—O72B—Mo8B—O82B	−0.1 (3)	Mo8B—O84B—Mo2B—O23B	77.7 (3)
Mo1B—O72B—Mo8B—O82B	−94.33 (19)	Mo8B—O84B—Mo2B—O13B	−77.6 (3)
Mo3B—O72B—Mo8B—O82B	93.46 (17)	Mo8B—O84B—Mo2B—O72B	−0.6 (2)
Mo2B—O72B—Mo8B—O82B	178.99 (13)	Mo7B—O72B—Mo2B—O22B	−174.2 (8)
Mo5B—O53B—Mo6B—O61B	178.7 (3)	Mo1B—O72B—Mo2B—O22B	−88.3 (8)
Mo5B—O53B—Mo6B—O62B	70.6 (3)	Mo8B—O72B—Mo2B—O22B	9.0 (8)
Mo5B—O53B—Mo6B—O83B	−48.3 (5)	Mo3B—O72B—Mo2B—O22B	105.4 (7)
Mo5B—O53B—Mo6B—O63B	−92.1 (3)	Mo7B—O72B—Mo2B—O21B	−2.6 (10)
Mo5B—O53B—Mo6B—O82B	−20.6 (3)	Mo1B—O72B—Mo2B—O21B	83.3 (2)
Mo8B—O83B—Mo6B—O61B	−178.0 (3)	Mo8B—O72B—Mo2B—O21B	−179.4 (2)
Mo1B—O83B—Mo6B—O61B	64.4 (3)	Mo3B—O72B—Mo2B—O21B	−83.0 (2)
Mo8B—O83B—Mo6B—O62B	−71.2 (3)	Mo7B—O72B—Mo2B—O23B	94.5 (10)
Mo1B—O83B—Mo6B—O62B	171.2 (3)	Mo1B—O72B—Mo2B—O23B	−179.58 (19)
Mo8B—O83B—Mo6B—O53B	48.7 (4)	Mo8B—O72B—Mo2B—O23B	−82.3 (2)
Mo1B—O83B—Mo6B—O53B	−68.9 (4)	Mo3B—O72B—Mo2B—O23B	14.12 (18)
Mo8B—O83B—Mo6B—O63B	95.1 (2)	Mo7B—O72B—Mo2B—O13B	−99.7 (10)
Mo1B—O83B—Mo6B—O63B	−22.46 (16)	Mo1B—O72B—Mo2B—O13B	−13.80 (16)
Mo8B—O83B—Mo6B—O82B	20.17 (19)	Mo8B—O72B—Mo2B—O13B	83.52 (19)
Mo1B—O83B—Mo6B—O82B	−97.4 (2)	Mo3B—O72B—Mo2B—O13B	179.9 (2)
Mo7B—O63B—Mo6B—O61B	167.3 (3)	Mo7B—O72B—Mo2B—O84B	177.2 (10)
Mo1B—O63B—Mo6B—O61B	−75.0 (3)	Mo1B—O72B—Mo2B—O84B	−96.90 (18)
Mo7B—O63B—Mo6B—O62B	−40.3 (9)	Mo8B—O72B—Mo2B—O84B	0.42 (15)
Mo1B—O63B—Mo6B—O62B	77.4 (8)	Mo3B—O72B—Mo2B—O84B	96.79 (17)
Mo7B—O63B—Mo6B—O53B	64.4 (2)	Mo5A—O53A—Mo6A—O61A	177.8 (3)
Mo1B—O63B—Mo6B—O53B	−177.9 (2)	Mo5A—O53A—Mo6A—O62A	69.7 (3)
Mo7B—O63B—Mo6B—O83B	−91.2 (3)	Mo5A—O53A—Mo6A—O83A	−49.7 (5)
Mo1B—O63B—Mo6B—O83B	26.5 (2)	Mo5A—O53A—Mo6A—O63A	−93.6 (3)
Mo7B—O63B—Mo6B—O82B	−14.4 (2)	Mo5A—O53A—Mo6A—O82A	−21.3 (3)
Mo1B—O63B—Mo6B—O82B	103.3 (2)	Mo8A—O83A—Mo6A—O61A	−177.4 (3)
Mo8B—O82B—Mo6B—O61B	−88.8 (7)	Mo1A—O83A—Mo6A—O61A	64.7 (3)
Mo4B—O82B—Mo6B—O61B	163.6 (8)	Mo8A—O83A—Mo6A—O62A	−71.1 (3)
Mo7B—O82B—Mo6B—O61B	16.2 (8)	Mo1A—O83A—Mo6A—O62A	171.0 (2)
Mo5B—O82B—Mo6B—O61B	106.8 (7)	Mo8A—O83A—Mo6A—O53A	49.7 (4)
Mo8B—O82B—Mo6B—O62B	78.5 (2)	Mo1A—O83A—Mo6A—O53A	−68.2 (4)
Mo4B—O82B—Mo6B—O62B	−29.2 (9)	Mo8A—O83A—Mo6A—O63A	96.2 (2)
Mo7B—O82B—Mo6B—O62B	−176.6 (2)	Mo1A—O83A—Mo6A—O63A	−21.72 (17)
Mo5B—O82B—Mo6B—O62B	−85.9 (2)	Mo8A—O83A—Mo6A—O82A	20.49 (19)
Mo8B—O82B—Mo6B—O53B	178.6 (2)	Mo1A—O83A—Mo6A—O82A	−97.4 (2)
Mo4B—O82B—Mo6B—O53B	70.9 (9)	Mo7A—O63A—Mo6A—O61A	166.9 (3)
Mo7B—O82B—Mo6B—O53B	−76.5 (2)	Mo1A—O63A—Mo6A—O61A	−75.9 (2)
Mo5B—O82B—Mo6B—O53B	14.16 (18)	Mo7A—O63A—Mo6A—O62A	−45.3 (8)
Mo8B—O82B—Mo6B—O83B	−17.50 (16)	Mo1A—O63A—Mo6A—O62A	71.9 (8)
Mo4B—O82B—Mo6B—O83B	−125.2 (9)	Mo7A—O63A—Mo6A—O53A	64.2 (2)
Mo7B—O82B—Mo6B—O83B	87.43 (18)	Mo1A—O63A—Mo6A—O53A	−178.6 (2)
Mo5B—O82B—Mo6B—O83B	178.07 (19)	Mo7A—O63A—Mo6A—O83A	−92.0 (2)
Mo8B—O82B—Mo6B—O63B	−93.86 (18)	Mo1A—O63A—Mo6A—O83A	25.1 (2)
Mo4B—O82B—Mo6B—O63B	158.5 (9)	Mo7A—O63A—Mo6A—O82A	−14.9 (2)
Mo7B—O82B—Mo6B—O63B	11.07 (16)	Mo1A—O63A—Mo6A—O82A	102.2 (2)
Mo5B—O82B—Mo6B—O63B	101.71 (17)	Mo8A—O82A—Mo6A—O61A	−87.8 (7)
Mo2A—O84A—Mo8A—O81A	−179.7 (3)	Mo4A—O82A—Mo6A—O61A	163.6 (8)
Mo2A—O84A—Mo8A—O33A	−76.4 (3)	Mo7A—O82A—Mo6A—O61A	17.4 (8)
Mo2A—O84A—Mo8A—O83A	76.9 (3)	Mo5A—O82A—Mo6A—O61A	108.2 (7)
Mo2A—O84A—Mo8A—O82A	0.5 (7)	Mo8A—O82A—Mo6A—O62A	77.6 (2)
Mo2A—O84A—Mo8A—O72A	0.9 (2)	Mo4A—O82A—Mo6A—O62A	−31.0 (9)
Mo4A—O33A—Mo8A—O81A	−76.3 (3)	Mo7A—O82A—Mo6A—O62A	−177.2 (2)
Mo3A—O33A—Mo8A—O81A	171.2 (2)	Mo5A—O82A—Mo6A—O62A	−86.4 (2)
Mo4A—O33A—Mo8A—O84A	177.2 (2)	Mo8A—O82A—Mo6A—O53A	178.4 (2)
Mo3A—O33A—Mo8A—O84A	64.7 (2)	Mo4A—O82A—Mo6A—O53A	69.9 (9)
Mo4A—O33A—Mo8A—O83A	62.3 (5)	Mo7A—O82A—Mo6A—O53A	−76.3 (2)
Mo3A—O33A—Mo8A—O83A	−50.2 (5)	Mo5A—O82A—Mo6A—O53A	14.48 (18)
Mo4A—O33A—Mo8A—O82A	21.4 (2)	Mo8A—O82A—Mo6A—O83A	−17.58 (16)
Mo3A—O33A—Mo8A—O82A	−91.1 (2)	Mo4A—O82A—Mo6A—O83A	−126.1 (9)
Mo4A—O33A—Mo8A—O72A	98.7 (2)	Mo7A—O82A—Mo6A—O83A	87.69 (19)
Mo3A—O33A—Mo8A—O72A	−13.83 (19)	Mo5A—O82A—Mo6A—O83A	178.5 (2)
Mo6A—O83A—Mo8A—O81A	75.6 (3)	Mo8A—O82A—Mo6A—O63A	−93.71 (18)
Mo1A—O83A—Mo8A—O81A	−170.9 (2)	Mo4A—O82A—Mo6A—O63A	157.7 (9)
Mo6A—O83A—Mo8A—O84A	−177.9 (2)	Mo7A—O82A—Mo6A—O63A	11.56 (16)
Mo1A—O83A—Mo8A—O84A	−64.5 (3)	Mo5A—O82A—Mo6A—O63A	102.35 (17)
Mo6A—O83A—Mo8A—O33A	−62.9 (4)	Mo5A—O43A—Mo4A—O41A	−179.8 (3)
Mo1A—O83A—Mo8A—O33A	50.5 (5)	Mo5A—O43A—Mo4A—O42A	−71.6 (3)
Mo6A—O83A—Mo8A—O82A	−22.0 (2)	Mo5A—O43A—Mo4A—O33A	47.7 (5)
Mo1A—O83A—Mo8A—O82A	91.4 (2)	Mo5A—O43A—Mo4A—O73A	92.2 (3)
Mo6A—O83A—Mo8A—O72A	−99.5 (2)	Mo5A—O43A—Mo4A—O82A	20.3 (2)
Mo1A—O83A—Mo8A—O72A	13.94 (19)	Mo8A—O33A—Mo4A—O41A	179.1 (3)
Mo4A—O82A—Mo8A—O81A	82.2 (2)	Mo3A—O33A—Mo4A—O41A	−63.8 (2)
Mo6A—O82A—Mo8A—O81A	−82.1 (2)	Mo8A—O33A—Mo4A—O42A	72.5 (3)
Mo7A—O82A—Mo8A—O81A	−179.6 (2)	Mo3A—O33A—Mo4A—O42A	−170.5 (2)
Mo5A—O82A—Mo8A—O81A	−1.7 (10)	Mo8A—O33A—Mo4A—O43A	−48.2 (5)
Mo4A—O82A—Mo8A—O84A	−98.1 (5)	Mo3A—O33A—Mo4A—O43A	68.9 (4)
Mo6A—O82A—Mo8A—O84A	97.7 (5)	Mo8A—O33A—Mo4A—O73A	−95.4 (2)
Mo7A—O82A—Mo8A—O84A	0.2 (6)	Mo3A—O33A—Mo4A—O73A	21.62 (17)
Mo5A—O82A—Mo8A—O84A	178.1 (7)	Mo8A—O33A—Mo4A—O82A	−20.08 (19)
Mo4A—O82A—Mo8A—O33A	−17.34 (17)	Mo3A—O33A—Mo4A—O82A	97.0 (2)
Mo6A—O82A—Mo8A—O33A	178.4 (2)	Mo7A—O73A—Mo4A—O41A	−165.4 (3)
Mo7A—O82A—Mo8A—O33A	80.9 (2)	Mo3A—O73A—Mo4A—O41A	76.1 (3)
Mo5A—O82A—Mo8A—O33A	−101.2 (9)	Mo7A—O73A—Mo4A—O42A	47.9 (8)
Mo4A—O82A—Mo8A—O83A	−178.1 (2)	Mo3A—O73A—Mo4A—O42A	−70.6 (8)
Mo6A—O82A—Mo8A—O83A	17.63 (16)	Mo7A—O73A—Mo4A—O43A	−63.8 (3)
Mo7A—O82A—Mo8A—O83A	−79.9 (2)	Mo3A—O73A—Mo4A—O43A	177.7 (2)
Mo5A—O82A—Mo8A—O83A	98.0 (10)	Mo7A—O73A—Mo4A—O33A	92.9 (3)
Mo4A—O82A—Mo8A—O72A	−98.41 (16)	Mo3A—O73A—Mo4A—O33A	−25.5 (2)
Mo6A—O82A—Mo8A—O72A	97.33 (15)	Mo7A—O73A—Mo4A—O82A	15.4 (2)
Mo7A—O82A—Mo8A—O72A	−0.1 (2)	Mo3A—O73A—Mo4A—O82A	−103.1 (2)
Mo5A—O82A—Mo8A—O72A	177.7 (9)	Mo8A—O82A—Mo4A—O41A	91.0 (7)
Mo7A—O72A—Mo8A—O81A	7(3)	Mo6A—O82A—Mo4A—O41A	−160.5 (8)
Mo1A—O72A—Mo8A—O81A	−88 (3)	Mo7A—O82A—Mo4A—O41A	−14.4 (7)
Mo3A—O72A—Mo8A—O81A	101 (3)	Mo5A—O82A—Mo4A—O41A	−105.2 (6)
Mo2A—O72A—Mo8A—O81A	−174 (3)	Mo8A—O82A—Mo4A—O42A	−77.4 (2)
Mo7A—O72A—Mo8A—O84A	−179.7 (2)	Mo6A—O82A—Mo4A—O42A	31.1 (9)
Mo1A—O72A—Mo8A—O84A	85.6 (2)	Mo7A—O82A—Mo4A—O42A	177.2 (2)
Mo3A—O72A—Mo8A—O84A	−85.8 (2)	Mo5A—O82A—Mo4A—O42A	86.4 (2)
Mo2A—O72A—Mo8A—O84A	−0.69 (18)	Mo8A—O82A—Mo4A—O43A	−177.7 (2)
Mo7A—O72A—Mo8A—O33A	−81.0 (2)	Mo6A—O82A—Mo4A—O43A	−69.2 (9)
Mo1A—O72A—Mo8A—O33A	−175.7 (2)	Mo7A—O82A—Mo4A—O43A	76.9 (2)
Mo3A—O72A—Mo8A—O33A	12.98 (18)	Mo5A—O82A—Mo4A—O43A	−13.91 (16)
Mo2A—O72A—Mo8A—O33A	98.06 (19)	Mo8A—O82A—Mo4A—O33A	17.22 (17)
Mo7A—O72A—Mo8A—O83A	81.6 (2)	Mo6A—O82A—Mo4A—O33A	125.7 (9)
Mo1A—O72A—Mo8A—O83A	−13.13 (17)	Mo7A—O82A—Mo4A—O33A	−88.1 (2)
Mo3A—O72A—Mo8A—O83A	175.5 (2)	Mo5A—O82A—Mo4A—O33A	−179.0 (2)
Mo2A—O72A—Mo8A—O83A	−99.39 (19)	Mo8A—O82A—Mo4A—O73A	93.43 (19)
Mo7A—O72A—Mo8A—O82A	0.2 (3)	Mo6A—O82A—Mo4A—O73A	−158.1 (9)
Mo1A—O72A—Mo8A—O82A	−94.56 (19)	Mo7A—O82A—Mo4A—O73A	−11.92 (15)
Mo3A—O72A—Mo8A—O82A	94.09 (18)	Mo5A—O82A—Mo4A—O73A	−102.75 (18)
Mo2A—O72A—Mo8A—O82A	179.17 (13)	Mo6A—O53A—Mo5A—O52A	−177.1 (3)
Mo5A—O74A—Mo7A—O71A	179.5 (3)	Mo6A—O53A—Mo5A—O51A	−70.8 (3)
Mo5A—O74A—Mo7A—O63A	75.8 (3)	Mo6A—O53A—Mo5A—O43A	45.6 (5)
Mo5A—O74A—Mo7A—O73A	−76.8 (3)	Mo6A—O53A—Mo5A—O74A	92.1 (3)
Mo5A—O74A—Mo7A—O72A	−0.5 (7)	Mo6A—O53A—Mo5A—O82A	20.5 (2)
Mo5A—O74A—Mo7A—O82A	−0.8 (2)	Mo4A—O43A—Mo5A—O52A	177.8 (3)
Mo1A—O63A—Mo7A—O71A	75.8 (3)	Mo4A—O43A—Mo5A—O51A	72.0 (3)
Mo6A—O63A—Mo7A—O71A	−170.7 (2)	Mo4A—O43A—Mo5A—O53A	−44.5 (5)
Mo1A—O63A—Mo7A—O74A	−178.4 (2)	Mo4A—O43A—Mo5A—O74A	−91.0 (3)
Mo6A—O63A—Mo7A—O74A	−64.9 (2)	Mo4A—O43A—Mo5A—O82A	−19.4 (2)
Mo1A—O63A—Mo7A—O73A	−62.7 (4)	Mo7A—O74A—Mo5A—O52A	179.7 (3)
Mo6A—O63A—Mo7A—O73A	50.8 (4)	Mo7A—O74A—Mo5A—O51A	1.2 (9)
Mo1A—O63A—Mo7A—O72A	−21.56 (19)	Mo7A—O74A—Mo5A—O53A	−76.8 (3)
Mo6A—O63A—Mo7A—O72A	91.9 (2)	Mo7A—O74A—Mo5A—O43A	77.9 (3)
Mo1A—O63A—Mo7A—O82A	−99.0 (2)	Mo7A—O74A—Mo5A—O82A	0.8 (2)
Mo6A—O63A—Mo7A—O82A	14.46 (19)	Mo8A—O82A—Mo5A—O52A	177.8 (8)
Mo3A—O73A—Mo7A—O71A	−75.2 (3)	Mo4A—O82A—Mo5A—O52A	92.9 (8)
Mo4A—O73A—Mo7A—O71A	170.3 (2)	Mo6A—O82A—Mo5A—O52A	−100.7 (8)
Mo3A—O73A—Mo7A—O74A	179.1 (2)	Mo7A—O82A—Mo5A—O52A	−4.3 (8)
Mo4A—O73A—Mo7A—O74A	64.6 (3)	Mo8A—O82A—Mo5A—O51A	1.7 (10)
Mo3A—O73A—Mo7A—O63A	63.3 (4)	Mo4A—O82A—Mo5A—O51A	−83.2 (2)
Mo4A—O73A—Mo7A—O63A	−51.2 (5)	Mo6A—O82A—Mo5A—O51A	83.1 (2)
Mo3A—O73A—Mo7A—O72A	22.24 (19)	Mo7A—O82A—Mo5A—O51A	179.6 (2)
Mo4A—O73A—Mo7A—O72A	−92.3 (2)	Mo8A—O82A—Mo5A—O53A	−96.0 (9)
Mo3A—O73A—Mo7A—O82A	99.8 (2)	Mo4A—O82A—Mo5A—O53A	179.2 (2)
Mo4A—O73A—Mo7A—O82A	−14.75 (19)	Mo6A—O82A—Mo5A—O53A	−14.48 (18)
Mo1A—O72A—Mo7A—O71A	−82.4 (2)	Mo7A—O82A—Mo5A—O53A	82.0 (2)
Mo3A—O72A—Mo7A—O71A	82.3 (2)	Mo8A—O82A—Mo5A—O43A	98.8 (10)
Mo8A—O72A—Mo7A—O71A	179.5 (2)	Mo4A—O82A—Mo5A—O43A	13.90 (16)
Mo2A—O72A—Mo7A—O71A	3.3 (10)	Mo6A—O82A—Mo5A—O43A	−179.7 (2)
Mo1A—O72A—Mo7A—O74A	97.7 (5)	Mo7A—O82A—Mo5A—O43A	−83.29 (19)
Mo3A—O72A—Mo7A—O74A	−97.7 (5)	Mo8A—O82A—Mo5A—O74A	−178.5 (10)
Mo8A—O72A—Mo7A—O74A	−0.4 (6)	Mo4A—O82A—Mo5A—O74A	96.67 (19)
Mo2A—O72A—Mo7A—O74A	−176.6 (7)	Mo6A—O82A—Mo5A—O74A	−96.98 (18)
Mo1A—O72A—Mo7A—O63A	17.57 (16)	Mo7A—O82A—Mo5A—O74A	−0.52 (16)
Mo3A—O72A—Mo7A—O63A	−177.78 (19)	Mo5B—O43B—Mo4B—O41B	180.0 (3)
Mo8A—O72A—Mo7A—O63A	−80.5 (2)	Mo5B—O43B—Mo4B—O42B	−71.7 (3)
Mo2A—O72A—Mo7A—O63A	103.2 (9)	Mo5B—O43B—Mo4B—O33B	49.2 (5)
Mo1A—O72A—Mo7A—O73A	177.70 (19)	Mo5B—O43B—Mo4B—O73B	92.7 (3)
Mo3A—O72A—Mo7A—O73A	−17.64 (15)	Mo5B—O43B—Mo4B—O82B	20.7 (2)
Mo8A—O72A—Mo7A—O73A	79.60 (19)	Mo8B—O33B—Mo4B—O41B	179.5 (3)
Mo2A—O72A—Mo7A—O73A	−96.6 (10)	Mo3B—O33B—Mo4B—O41B	−62.4 (2)
Mo1A—O72A—Mo7A—O82A	97.96 (16)	Mo8B—O33B—Mo4B—O42B	72.9 (3)
Mo3A—O72A—Mo7A—O82A	−97.39 (15)	Mo3B—O33B—Mo4B—O42B	−169.1 (2)
Mo8A—O72A—Mo7A—O82A	−0.1 (2)	Mo8B—O33B—Mo4B—O43B	−49.5 (4)
Mo2A—O72A—Mo7A—O82A	−176.4 (9)	Mo3B—O33B—Mo4B—O43B	68.5 (4)
Mo8A—O82A—Mo7A—O71A	−4(3)	Mo8B—O33B—Mo4B—O73B	−95.6 (2)
Mo4A—O82A—Mo7A—O71A	91 (3)	Mo3B—O33B—Mo4B—O73B	22.40 (16)
Mo6A—O82A—Mo7A—O71A	−98 (3)	Mo8B—O33B—Mo4B—O82B	−20.18 (19)
Mo5A—O82A—Mo7A—O71A	177 (3)	Mo3B—O33B—Mo4B—O82B	97.8 (2)
Mo8A—O82A—Mo7A—O74A	−180.0 (3)	Mo7B—O73B—Mo4B—O41B	−166.5 (3)
Mo4A—O82A—Mo7A—O74A	−85.1 (2)	Mo3B—O73B—Mo4B—O41B	75.5 (2)
Mo6A—O82A—Mo7A—O74A	85.6 (2)	Mo7B—O73B—Mo4B—O42B	47.1 (8)
Mo5A—O82A—Mo7A—O74A	0.6 (2)	Mo3B—O73B—Mo4B—O42B	−70.9 (8)
Mo8A—O82A—Mo7A—O63A	81.1 (2)	Mo7B—O73B—Mo4B—O43B	−64.7 (3)
Mo4A—O82A—Mo7A—O63A	175.9 (2)	Mo3B—O73B—Mo4B—O43B	177.3 (2)
Mo6A—O82A—Mo7A—O63A	−13.35 (18)	Mo7B—O73B—Mo4B—O33B	91.6 (2)
Mo5A—O82A—Mo7A—O63A	−98.34 (19)	Mo3B—O73B—Mo4B—O33B	−26.4 (2)
Mo8A—O82A—Mo7A—O73A	−81.0 (2)	Mo7B—O73B—Mo4B—O82B	14.5 (2)
Mo4A—O82A—Mo7A—O73A	13.81 (17)	Mo3B—O73B—Mo4B—O82B	−103.5 (2)
Mo6A—O82A—Mo7A—O73A	−175.4 (2)	Mo8B—O82B—Mo4B—O41B	91.1 (6)
Mo5A—O82A—Mo7A—O73A	99.6 (2)	Mo7B—O82B—Mo4B—O41B	−14.0 (7)
Mo8A—O82A—Mo7A—O72A	0.2 (3)	Mo6B—O82B—Mo4B—O41B	−161.4 (7)
Mo4A—O82A—Mo7A—O72A	94.97 (19)	Mo5B—O82B—Mo4B—O41B	−104.7 (6)
Mo6A—O82A—Mo7A—O72A	−94.26 (17)	Mo8B—O82B—Mo4B—O42B	−77.4 (2)
Mo5A—O82A—Mo7A—O72A	−179.25 (13)	Mo7B—O82B—Mo4B—O42B	177.5 (2)
Mo5B—O74B—Mo7B—O71B	−179.8 (3)	Mo6B—O82B—Mo4B—O42B	30.2 (9)
Mo5B—O74B—Mo7B—O63B	76.0 (3)	Mo5B—O82B—Mo4B—O42B	86.8 (2)
Mo5B—O74B—Mo7B—O73B	−77.3 (3)	Mo8B—O82B—Mo4B—O43B	−178.6 (2)
Mo5B—O74B—Mo7B—O72B	−0.1 (7)	Mo7B—O82B—Mo4B—O43B	76.3 (2)
Mo5B—O74B—Mo7B—O82B	−0.8 (2)	Mo6B—O82B—Mo4B—O43B	−71.0 (8)
Mo1B—O63B—Mo7B—O71B	75.3 (3)	Mo5B—O82B—Mo4B—O43B	−14.36 (16)
Mo6B—O63B—Mo7B—O71B	−170.5 (2)	Mo8B—O82B—Mo4B—O33B	17.57 (17)
Mo1B—O63B—Mo7B—O74B	−179.5 (2)	Mo7B—O82B—Mo4B—O33B	−87.5 (2)
Mo6B—O63B—Mo7B—O74B	−65.3 (2)	Mo6B—O82B—Mo4B—O33B	125.1 (9)
Mo1B—O63B—Mo7B—O73B	−65.0 (4)	Mo5B—O82B—Mo4B—O33B	−178.2 (2)
Mo6B—O63B—Mo7B—O73B	49.2 (5)	Mo8B—O82B—Mo4B—O73B	93.79 (19)
Mo1B—O63B—Mo7B—O72B	−22.4 (2)	Mo7B—O82B—Mo4B—O73B	−11.32 (15)
Mo6B—O63B—Mo7B—O72B	91.8 (2)	Mo6B—O82B—Mo4B—O73B	−158.7 (9)
Mo1B—O63B—Mo7B—O82B	−100.2 (2)	Mo5B—O82B—Mo4B—O73B	−102.02 (17)
Mo6B—O63B—Mo7B—O82B	14.1 (2)	Mo2A—O13A—Mo1A—O11A	180.0 (3)
Mo3B—O73B—Mo7B—O71B	−76.1 (3)	Mo2A—O13A—Mo1A—O12A	72.9 (3)
Mo4B—O73B—Mo7B—O71B	170.3 (2)	Mo2A—O13A—Mo1A—O63A	−47.6 (5)
Mo3B—O73B—Mo7B—O74B	178.9 (2)	Mo2A—O13A—Mo1A—O83A	−92.2 (3)
Mo4B—O73B—Mo7B—O74B	65.3 (2)	Mo2A—O13A—Mo1A—O72A	−19.5 (2)
Mo3B—O73B—Mo7B—O63B	64.5 (4)	Mo7A—O63A—Mo1A—O11A	−178.2 (2)
Mo4B—O73B—Mo7B—O63B	−49.1 (5)	Mo6A—O63A—Mo1A—O11A	63.6 (2)
Mo3B—O73B—Mo7B—O72B	21.96 (19)	Mo7A—O63A—Mo1A—O12A	−72.5 (3)
Mo4B—O73B—Mo7B—O72B	−91.7 (2)	Mo6A—O63A—Mo1A—O12A	169.3 (2)
Mo3B—O73B—Mo7B—O82B	99.7 (2)	Mo7A—O63A—Mo1A—O13A	49.1 (4)
Mo4B—O73B—Mo7B—O82B	−13.94 (19)	Mo6A—O63A—Mo1A—O13A	−69.1 (4)
Mo1B—O72B—Mo7B—O71B	−82.6 (2)	Mo7A—O63A—Mo1A—O83A	96.5 (2)
Mo8B—O72B—Mo7B—O71B	179.2 (2)	Mo6A—O63A—Mo1A—O83A	−21.72 (17)
Mo3B—O72B—Mo7B—O71B	82.0 (2)	Mo7A—O63A—Mo1A—O72A	20.40 (18)
Mo2B—O72B—Mo7B—O71B	2.5 (10)	Mo6A—O63A—Mo1A—O72A	−97.8 (2)
Mo1B—O72B—Mo7B—O74B	97.7 (5)	Mo8A—O83A—Mo1A—O11A	166.2 (3)
Mo8B—O72B—Mo7B—O74B	−0.6 (6)	Mo6A—O83A—Mo1A—O11A	−76.4 (2)
Mo3B—O72B—Mo7B—O74B	−97.7 (5)	Mo8A—O83A—Mo1A—O12A	−48.8 (9)
Mo2B—O72B—Mo7B—O74B	−177.2 (7)	Mo6A—O83A—Mo1A—O12A	68.6 (8)
Mo1B—O72B—Mo7B—O63B	17.98 (16)	Mo8A—O83A—Mo1A—O13A	64.2 (2)
Mo8B—O72B—Mo7B—O63B	−80.2 (2)	Mo6A—O83A—Mo1A—O13A	−178.4 (2)
Mo3B—O72B—Mo7B—O63B	−177.39 (19)	Mo8A—O83A—Mo1A—O63A	−92.2 (3)
Mo2B—O72B—Mo7B—O63B	103.1 (10)	Mo6A—O83A—Mo1A—O63A	25.15 (19)
Mo1B—O72B—Mo7B—O73B	178.23 (19)	Mo8A—O83A—Mo1A—O72A	−14.6 (2)
Mo8B—O72B—Mo7B—O73B	80.02 (19)	Mo6A—O83A—Mo1A—O72A	102.7 (2)
Mo3B—O72B—Mo7B—O73B	−17.14 (15)	Mo7A—O72A—Mo1A—O11A	−90.8 (7)
Mo2B—O72B—Mo7B—O73B	−96.7 (10)	Mo3A—O72A—Mo1A—O11A	161.3 (7)
Mo1B—O72B—Mo7B—O82B	98.32 (15)	Mo8A—O72A—Mo1A—O11A	13.8 (7)
Mo8B—O72B—Mo7B—O82B	0.1 (2)	Mo2A—O72A—Mo1A—O11A	104.4 (6)
Mo3B—O72B—Mo7B—O82B	−97.05 (15)	Mo7A—O72A—Mo1A—O12A	77.8 (2)
Mo2B—O72B—Mo7B—O82B	−176.6 (9)	Mo3A—O72A—Mo1A—O12A	−30.1 (9)
Mo8B—O82B—Mo7B—O71B	−12 (3)	Mo8A—O72A—Mo1A—O12A	−177.6 (2)
Mo4B—O82B—Mo7B—O71B	83 (3)	Mo2A—O72A—Mo1A—O12A	−87.0 (2)
Mo6B—O82B—Mo7B—O71B	−106 (3)	Mo7A—O72A—Mo1A—O13A	178.1 (2)
Mo5B—O82B—Mo7B—O71B	169 (3)	Mo3A—O72A—Mo1A—O13A	70.1 (9)
Mo8B—O82B—Mo7B—O74B	179.6 (3)	Mo8A—O72A—Mo1A—O13A	−77.3 (2)
Mo4B—O82B—Mo7B—O74B	−85.3 (2)	Mo2A—O72A—Mo1A—O13A	13.19 (16)
Mo6B—O82B—Mo7B—O74B	85.6 (2)	Mo7A—O72A—Mo1A—O63A	−17.38 (16)
Mo5B—O82B—Mo7B—O74B	0.65 (19)	Mo3A—O72A—Mo1A—O63A	−125.3 (9)
Mo8B—O82B—Mo7B—O63B	81.2 (2)	Mo8A—O72A—Mo1A—O63A	87.23 (19)
Mo4B—O82B—Mo7B—O63B	176.3 (2)	Mo2A—O72A—Mo1A—O63A	177.75 (18)
Mo6B—O82B—Mo7B—O63B	−12.77 (18)	Mo7A—O72A—Mo1A—O83A	−93.26 (18)
Mo5B—O82B—Mo7B—O63B	−97.7 (2)	Mo3A—O72A—Mo1A—O83A	158.8 (9)
Mo8B—O82B—Mo7B—O73B	−82.0 (2)	Mo8A—O72A—Mo1A—O83A	11.36 (15)
Mo4B—O82B—Mo7B—O73B	13.08 (17)	Mo2A—O72A—Mo1A—O83A	101.88 (17)
Mo6B—O82B—Mo7B—O73B	−176.0 (2)	Mo2B—O13B—Mo1B—O11B	179.4 (3)
Mo5B—O82B—Mo7B—O73B	99.08 (19)	Mo2B—O13B—Mo1B—O12B	72.1 (3)
Mo8B—O82B—Mo7B—O72B	−0.1 (3)	Mo2B—O13B—Mo1B—O63B	−48.8 (5)
Mo4B—O82B—Mo7B—O72B	94.92 (19)	Mo2B—O13B—Mo1B—O72B	−20.0 (2)
Mo6B—O82B—Mo7B—O72B	−94.13 (17)	Mo2B—O13B—Mo1B—O83B	−92.5 (3)
Mo5B—O82B—Mo7B—O72B	−179.08 (13)	Mo7B—O63B—Mo1B—O11B	−177.9 (2)
Mo6B—O53B—Mo5B—O52B	−177.4 (3)	Mo6B—O63B—Mo1B—O11B	62.8 (2)
Mo6B—O53B—Mo5B—O51B	−70.0 (3)	Mo7B—O63B—Mo1B—O12B	−72.0 (3)
Mo6B—O53B—Mo5B—O43B	45.1 (5)	Mo6B—O63B—Mo1B—O12B	168.7 (2)
Mo6B—O53B—Mo5B—O74B	92.4 (3)	Mo7B—O63B—Mo1B—O13B	50.3 (4)
Mo6B—O53B—Mo5B—O82B	20.2 (3)	Mo6B—O63B—Mo1B—O13B	−69.1 (4)
Mo4B—O43B—Mo5B—O52B	177.0 (3)	Mo7B—O63B—Mo1B—O72B	20.86 (19)
Mo4B—O43B—Mo5B—O51B	70.1 (3)	Mo6B—O63B—Mo1B—O72B	−98.5 (2)
Mo4B—O43B—Mo5B—O53B	−45.0 (5)	Mo7B—O63B—Mo1B—O83B	96.9 (2)
Mo4B—O43B—Mo5B—O74B	−92.5 (3)	Mo6B—O63B—Mo1B—O83B	−22.47 (16)
Mo4B—O43B—Mo5B—O82B	−20.0 (2)	Mo7B—O72B—Mo1B—O11B	−92.9 (7)
Mo7B—O74B—Mo5B—O52B	179.0 (3)	Mo8B—O72B—Mo1B—O11B	11.2 (7)
Mo7B—O74B—Mo5B—O51B	1.9 (9)	Mo3B—O72B—Mo1B—O11B	161.8 (8)
Mo7B—O74B—Mo5B—O53B	−77.2 (3)	Mo2B—O72B—Mo1B—O11B	102.6 (6)
Mo7B—O74B—Mo5B—O43B	77.9 (3)	Mo7B—O72B—Mo1B—O12B	77.6 (2)
Mo7B—O74B—Mo5B—O82B	0.8 (2)	Mo8B—O72B—Mo1B—O12B	−178.3 (2)
Mo8B—O82B—Mo5B—O52B	177.1 (8)	Mo3B—O72B—Mo1B—O12B	−27.7 (9)
Mo4B—O82B—Mo5B—O52B	90.8 (7)	Mo2B—O72B—Mo1B—O12B	−86.9 (2)
Mo7B—O82B—Mo5B—O52B	−6.7 (8)	Mo7B—O72B—Mo1B—O13B	178.6 (2)
Mo6B—O82B—Mo5B—O52B	−103.2 (7)	Mo8B—O72B—Mo1B—O13B	−77.4 (2)
Mo8B—O82B—Mo5B—O51B	3.5 (10)	Mo3B—O72B—Mo1B—O13B	73.3 (8)
Mo4B—O82B—Mo5B—O51B	−82.7 (2)	Mo2B—O72B—Mo1B—O13B	14.04 (16)
Mo7B—O82B—Mo5B—O51B	179.8 (2)	Mo7B—O72B—Mo1B—O63B	−17.91 (16)
Mo6B—O82B—Mo5B—O51B	83.2 (2)	Mo8B—O72B—Mo1B—O63B	86.15 (19)
Mo8B—O82B—Mo5B—O53B	−93.8 (10)	Mo3B—O72B—Mo1B—O63B	−123.2 (9)
Mo4B—O82B—Mo5B—O53B	180.0 (2)	Mo2B—O72B—Mo1B—O63B	177.56 (19)
Mo7B—O82B—Mo5B—O53B	82.5 (2)	Mo7B—O72B—Mo1B—O83B	−93.17 (19)
Mo6B—O82B—Mo5B—O53B	−14.07 (19)	Mo8B—O72B—Mo1B—O83B	10.88 (15)
Mo8B—O82B—Mo5B—O43B	100.4 (10)	Mo3B—O72B—Mo1B—O83B	161.6 (9)
Mo4B—O82B—Mo5B—O43B	14.18 (16)	Mo2B—O72B—Mo1B—O83B	102.30 (17)
Mo7B—O82B—Mo5B—O43B	−83.29 (19)	Mo8B—O83B—Mo1B—O11B	166.2 (3)
Mo6B—O82B—Mo5B—O43B	−179.9 (2)	Mo6B—O83B—Mo1B—O11B	−76.1 (3)
Mo8B—O82B—Mo5B—O74B	−176.8 (10)	Mo8B—O83B—Mo1B—O12B	−48.4 (9)
Mo4B—O82B—Mo5B—O74B	96.93 (18)	Mo6B—O83B—Mo1B—O12B	69.3 (8)
Mo7B—O82B—Mo5B—O74B	−0.54 (16)	Mo8B—O83B—Mo1B—O13B	64.4 (3)
Mo6B—O82B—Mo5B—O74B	−97.12 (18)	Mo6B—O83B—Mo1B—O13B	−178.0 (2)
Mo2A—O23A—Mo3A—O31A	−177.3 (3)	Mo8B—O83B—Mo1B—O63B	−91.3 (3)
Mo2A—O23A—Mo3A—O32A	−69.9 (3)	Mo6B—O83B—Mo1B—O63B	26.34 (19)
Mo2A—O23A—Mo3A—O73A	50.9 (5)	Mo8B—O83B—Mo1B—O72B	−13.9 (2)
Mo2A—O23A—Mo3A—O33A	93.9 (3)	Mo6B—O83B—Mo1B—O72B	103.8 (2)
Mo2A—O23A—Mo3A—O72A	21.1 (2)	C18B—C17B—N1B—C15B	56.3 (13)
Mo7A—O73A—Mo3A—O31A	176.3 (3)	C18B—C17B—N1B—C11B	177.0 (11)
Mo4A—O73A—Mo3A—O31A	−64.9 (3)	C18B—C17B—N1B—C13B	−62.3 (13)
Mo7A—O73A—Mo3A—O32A	70.2 (3)	C16B—C15B—N1B—C17B	51.9 (13)
Mo4A—O73A—Mo3A—O32A	−171.0 (2)	C16B—C15B—N1B—C11B	−64.8 (12)
Mo7A—O73A—Mo3A—O23A	−51.4 (4)	C16B—C15B—N1B—C13B	173.5 (10)
Mo4A—O73A—Mo3A—O23A	67.4 (4)	C12B—C11B—N1B—C17B	174.3 (10)
Mo7A—O73A—Mo3A—O33A	−97.0 (2)	C12B—C11B—N1B—C15B	−65.1 (12)
Mo4A—O73A—Mo3A—O33A	21.82 (17)	C12B—C11B—N1B—C13B	53.6 (13)
Mo7A—O73A—Mo3A—O72A	−20.76 (18)	C14B—C13B—N1B—C17B	−59.8 (11)
Mo4A—O73A—Mo3A—O72A	98.0 (2)	C14B—C13B—N1B—C15B	178.6 (9)
Mo8A—O33A—Mo3A—O31A	−167.5 (3)	C14B—C13B—N1B—C11B	56.9 (11)
Mo4A—O33A—Mo3A—O31A	75.9 (2)	C36A—C35A—N3A—C37A	−50.5 (12)
Mo8A—O33A—Mo3A—O32A	41.6 (9)	C36A—C35A—N3A—C33A	−171.7 (9)
Mo4A—O33A—Mo3A—O32A	−75.0 (8)	C36A—C35A—N3A—C31A	67.8 (11)
Mo8A—O33A—Mo3A—O23A	−64.9 (2)	C38A—C37A—N3A—C35A	−58.9 (10)
Mo4A—O33A—Mo3A—O23A	178.5 (2)	C38A—C37A—N3A—C33A	58.6 (10)
Mo8A—O33A—Mo3A—O73A	91.4 (3)	C38A—C37A—N3A—C31A	−179.5 (9)
Mo4A—O33A—Mo3A—O73A	−25.2 (2)	C34A—C33A—N3A—C35A	−177.7 (10)
Mo8A—O33A—Mo3A—O72A	14.4 (2)	C34A—C33A—N3A—C37A	60.4 (12)
Mo4A—O33A—Mo3A—O72A	−102.2 (2)	C34A—C33A—N3A—C31A	−58.3 (12)
Mo7A—O72A—Mo3A—O31A	87.3 (7)	C32A—C31A—N3A—C35A	58.3 (12)
Mo1A—O72A—Mo3A—O31A	−164.7 (8)	C32A—C31A—N3A—C37A	−179.6 (9)
Mo8A—O72A—Mo3A—O31A	−17.2 (8)	C32A—C31A—N3A—C33A	−58.0 (12)
Mo2A—O72A—Mo3A—O31A	−107.6 (7)	C38B—C37B—N3B—C33B	60.3 (12)
Mo7A—O72A—Mo3A—O32A	−79.1 (2)	C38B—C37B—N3B—C35B	−58.5 (12)
Mo1A—O72A—Mo3A—O32A	28.9 (9)	C38B—C37B—N3B—C31B	−177.6 (9)
Mo8A—O72A—Mo3A—O32A	176.4 (2)	C34B—C33B—N3B—C37B	61.5 (14)
Mo2A—O72A—Mo3A—O32A	86.0 (2)	C34B—C33B—N3B—C35B	−176.6 (12)
Mo7A—O72A—Mo3A—O23A	−179.4 (2)	C34B—C33B—N3B—C31B	−55.0 (15)
Mo1A—O72A—Mo3A—O23A	−71.4 (9)	C36B—C35B—N3B—C37B	−50.2 (16)
Mo8A—O72A—Mo3A—O23A	76.1 (2)	C36B—C35B—N3B—C33B	−171.4 (12)
Mo2A—O72A—Mo3A—O23A	−14.33 (17)	C36B—C35B—N3B—C31B	65.1 (15)
Mo7A—O72A—Mo3A—O73A	17.58 (16)	C32B—C31B—N3B—C37B	−174.9 (11)
Mo1A—O72A—Mo3A—O73A	125.6 (9)	C32B—C31B—N3B—C33B	−54.6 (15)
Mo8A—O72A—Mo3A—O73A	−86.90 (18)	C32B—C31B—N3B—C35B	64.9 (13)
Mo2A—O72A—Mo3A—O73A	−177.34 (19)	C22B—C21B—N2B—C27B	57.7 (12)
Mo7A—O72A—Mo3A—O33A	93.35 (18)	C22B—C21B—N2B—C23B	−179.5 (9)
Mo1A—O72A—Mo3A—O33A	−158.6 (9)	C22B—C21B—N2B—C25B	−61.1 (11)
Mo8A—O72A—Mo3A—O33A	−11.13 (16)	C28B—C27B—N2B—C21B	57.7 (12)
Mo2A—O72A—Mo3A—O33A	−101.57 (17)	C28B—C27B—N2B—C23B	−61.8 (11)
Mo2B—O23B—Mo3B—O31B	−178.8 (3)	C28B—C27B—N2B—C25B	178.1 (10)
Mo2B—O23B—Mo3B—O32B	−70.6 (3)	C24B—C23B—N2B—C21B	178.0 (9)
Mo2B—O23B—Mo3B—O73B	47.8 (5)	C24B—C23B—N2B—C27B	−58.4 (11)
Mo2B—O23B—Mo3B—O33B	92.7 (3)	C24B—C23B—N2B—C25B	59.8 (11)
Mo2B—O23B—Mo3B—O72B	20.5 (2)	C26B—C25B—N2B—C21B	−59.6 (11)
Mo7B—O73B—Mo3B—O31B	178.1 (2)	C26B—C25B—N2B—C27B	177.6 (9)
Mo4B—O73B—Mo3B—O31B	−63.7 (2)	C26B—C25B—N2B—C23B	56.3 (10)
Mo7B—O73B—Mo3B—O32B	71.5 (3)	C28A—C27A—N2A—C21A	−58.0 (10)
Mo4B—O73B—Mo3B—O32B	−170.4 (2)	C28A—C27A—N2A—C23A	59.8 (10)
Mo7B—O73B—Mo3B—O23B	−48.0 (4)	C28A—C27A—N2A—C25A	−179.5 (9)
Mo4B—O73B—Mo3B—O23B	70.1 (4)	C22A—C21A—N2A—C27A	−56.8 (11)
Mo7B—O73B—Mo3B—O33B	−95.7 (2)	C22A—C21A—N2A—C23A	−177.4 (9)
Mo4B—O73B—Mo3B—O33B	22.49 (17)	C22A—C21A—N2A—C25A	61.9 (11)
Mo7B—O73B—Mo3B—O72B	−20.11 (18)	C24A—C23A—N2A—C27A	57.0 (11)
Mo4B—O73B—Mo3B—O72B	98.1 (2)	C24A—C23A—N2A—C21A	179.1 (9)
Mo8B—O33B—Mo3B—O31B	−167.8 (3)	C24A—C23A—N2A—C25A	−60.5 (11)
Mo4B—O33B—Mo3B—O31B	75.4 (2)	C26A—C25A—N2A—C27A	177.8 (10)
Mo8B—O33B—Mo3B—O32B	41.5 (9)	C26A—C25A—N2A—C21A	55.0 (12)
Mo4B—O33B—Mo3B—O32B	−75.3 (8)	C26A—C25A—N2A—C23A	−62.1 (12)
Mo8B—O33B—Mo3B—O23B	−64.1 (2)	C18A—C17A—N1A—C15A	56.4 (16)
Mo4B—O33B—Mo3B—O23B	179.0 (2)	C18A—C17A—N1A—C13A	−62.4 (16)
Mo8B—O33B—Mo3B—O73B	90.5 (2)	C18A—C17A—N1A—C11A	176.3 (13)
Mo4B—O33B—Mo3B—O73B	−26.3 (2)	C16A—C15A—N1A—C17A	52.0 (16)
Mo8B—O33B—Mo3B—O72B	13.6 (2)	C16A—C15A—N1A—C13A	176.7 (12)
Mo4B—O33B—Mo3B—O72B	−103.2 (2)	C16A—C15A—N1A—C11A	−63.7 (14)
Mo7B—O72B—Mo3B—O31B	88.9 (7)	C14A—C13A—N1A—C17A	−59.1 (14)
Mo1B—O72B—Mo3B—O31B	−165.8 (8)	C14A—C13A—N1A—C15A	178.6 (10)
Mo8B—O72B—Mo3B—O31B	−15.0 (7)	C14A—C13A—N1A—C11A	58.6 (12)
Mo2B—O72B—Mo3B—O31B	−106.4 (6)	C12A—C11A—N1A—C17A	−179.2 (12)
Mo7B—O72B—Mo3B—O32B	−78.8 (2)	C12A—C11A—N1A—C15A	−59.4 (14)
Mo1B—O72B—Mo3B—O32B	26.6 (9)	C12A—C11A—N1A—C13A	56.7 (15)

### Hydrogen-bond geometry (Å, °)

**Table:**

*D*—H···*A*	*D*—H	H···*A*	*D*···*A*	*D*—H···*A*
N4A—H1N···O12B	0.82	2.399 (6)	2.908 (10)	121.32 (58)
N4A—H1N···O21B	0.82	2.388 (5)	2.851 (10)	116.76 (59)
N4A—H1N···O32B	0.82	2.571 (5)	2.936 (9)	108.68 (57)
N4A—H1N···O71B	0.82	2.482 (5)	2.904 (10)	113.29 (55)
N4A—H2N···O42A	0.96	2.457 (5)	2.856 (10)	104.87 (51)
N4A—H2N···O51A	0.96	2.216 (5)	2.915 (10)	129.21 (53)
N4A—H3N···O12B	0.73	2.566 (6)	2.908 (10)	110.92 (67)
N4A—H3N···O62A	0.73	2.261 (5)	2.909 (9)	148.30 (5)
N4A—H4N···O42A	0.96	2.511 (6)	2.856 (10)	101.14 (51)
N4A—H4N···O81A	0.96	1.999 (5)	2.865 (10)	149.24 (50)
N4B—H5N···O12A^i^	0.84	2.441 (6)	2.854 (10)	110.89 (56)
N4B—H5N···O32A^i^	0.84	2.445 (5)	2.950 (9)	119.12 (54)
N4B—H5N···O71A^i^	0.84	2.458 (6)	2.943 (10)	117.38 (53)
N4B—H6N···O12A^i^	0.95	2.387 (5)	2.854 (10)	109.94 (50)
N4B—H6N···O62B	0.95	2.214 (5)	2.992 (9)	138.49 (51)
N4B—H7N···O42B	0.97	2.385 (6)	2.820 (10)	106.78 (50)
N4B—H7N···O81B	0.97	2.089 (5)	2.895 (10)	139.63 (48)
N4B—H8N···O51B	0.96	2.122 (5)	2.855 (10)	132.53 (52)

Symmetry codes:  (i) *x*, *y*, *z*−1.
